# A Review on Polyacrylonitrile as an Effective and Economic Constituent of Adsorbents for Wastewater Treatment

**DOI:** 10.3390/molecules27248689

**Published:** 2022-12-08

**Authors:** Archana Gupta, Vishal Sharma, Pawan Kumar Mishra, Adam Ekielski

**Affiliations:** 1 P.G. Department of Chemistry, Mehr Chand Mahajan DAV College for Women, Sector 36-A, Chandigarh 160036, India; 2 Institute of Forensic Science and Criminology, Panjab University, Chandigarh 160014, India; 3 Faculty of Business and Economics, Mendel University in Brno, 61300 Brno, Czech Republic; 4 Department of Production Engineering, Warsaw University of Life Sciences, 02-776 Warsaw, Poland

**Keywords:** polyacrylonitrile, adsorbent, wastewater treatment, heavy metal, dyes

## Abstract

Water gets polluted due to the dumping of untreated industrial waste into bodies of water, particularly those containing heavy metals and dyes. Industrial water contains both inorganic and organic wastes. Numerous adsorbents that are inexpensive and easily available can be used to address the issue of water deterioration. This review report is focused on polyacrylonitrile as an efficient constituent of adsorbents to extract toxic ions and dyes. It discusses the various formulations of polyacrylonitrile, such as ion exchange resins, chelating resins, fibers, membranes, and hydrogels, synthesized through different polymerization methods, such as suspension polymerization, electrospinning, grafting, redox, and emulsion polymerization. Moreover, regeneration of adsorbent and heavy metal ions makes the adsorption process more cost-effective and efficient. The literature reporting successful regeneration of the adsorbent is included. The factors affecting the performance and outcomes of the adsorption process are also discussed.

## 1. Introduction

Safe and readily accessible water is vital for everyone, whether it is used for drinking, domestic use, food production, or amusement purposes. Better water supply and more hygienic and improved management of water resources can improve countries’ state of health and living. A shocking increase in toxic metal contaminants over the last few decades has caused several problems for biotic organisms and ecology [1]. The harmful contaminants in the water have detrimental effects on marine life and make the water unsafe for consumption by humans. Polluted water contains bacteria which cause diseases such as diarrhea, cholera, dysentery, typhoid, hepatitis A, and polio [2,3].

Various research activities have been conducted to generate techniques for extracting toxic ions and dyes, especially in the aqueous solution of hydrometallurgy, and hence to reutilize these ions. The methods of removing metal ions include filtration, chemical precipitation, electrocoagulation, neutralization, chelating ion exchange, and adsorption [4,5,6,7,8]. A volume of work has been conducted by researchers to extract these ions by utilizing various techniques and different low-cost adsorbents such as lignin, chitosan, clay, zeolite, activated carbon, polymeric adsorbents, chelating resins, etc. [9]. It is found from the literature that adsorption is the most favored method due to its simplicity, easy accessibility, cost efficacy, selectivity, and efficiency for the extraction of heavy toxic metal ions, dyes, and other pollutants [10,11]. According to the literature, a chelating resin, compared with traditional anion and cation exchange resins, is more effective in removing pollutants and also overcomes the low selectivity of ion exchange resins [12,13,14]. Generally, adsorption of toxic ions is done by moieties present on the surface of the adsorbent [15,16]. Moreover, chelating adsorbents can be produced by modifying the polymers with functional groups [17]. Chelating resins adsorb the toxic wastes present in the water on their surface through various chemical and physical attractions. New classes of synthetic permeable polymeric adsorbents have been discovered with adequate porosity, ample surface area, greater structural stability, and quick adsorption kinetics. A chelating agent is a type of ion exchange resin that is highly selective for anions or clusters of ions. They are capable of adsorbing ions through coordination interaction as a substitute for columbic forces in ion exchangers. Several chelating resins mentioned in published studies are synthesized by modifying polyacrylonitrile with different ligands such as polyethylene imine, amidoxime, acrylamide, dithiocarbonate, thiazoline, and amino acids [18].

**Figure 1:**
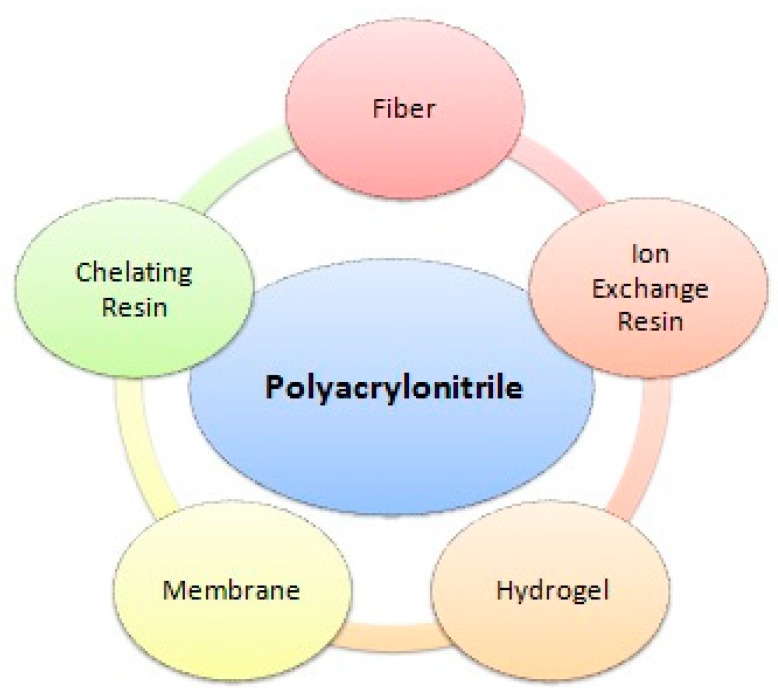
Use of polyacrylonitrile in different forms for extraction of metal ions.

Although there are several different composites and conjugates used for wastewater treatment, polyacrylonitrile has attracted great interest because of its exceptional properties such as environmental compatibility, commercial feasibility, high temperature resistance, and high tensile strength [19]. Polyacrylonitrile is an inexpensive raw substance utilized in fabric production units and is used in ion exchange substances in the form of beads due to their high porosity. Different varieties of polyacrylonitrile adsorbents are synthesized by mixing various monomers with acrylonitrile as a basic unit. It is a multipurpose polymer utilized to fabricate various products, including ultrafiltration membranes, hydrogels, hollow fibers for reverse osmosis, fibers for textiles, oxidized polyacrylonitrile fibers, etc. (Figure 1).

Polyacrylonitrile fibers are the chemical initiators to form carbon fibers with good characteristics [11]. Among the various polymers used for toxic pollutant removal, polyacrylonitrile is highlighted in this paper because it contains a nitrile group (-CN) and can easily form different stable products after functionalization with other groups. 

There are many reports published on polyacrylonitrile for wastewater treatment; however, most of them are either focused on fiber forms of polyacrylonitrile, or do not comprehensively cover all forms [20,21,22,23]. The purpose of this review is to investigate in one place all the various formulations of polyacrylonitrile as valuable adsorbents for the extraction of water pollutants, along with the factors that alter the performance of the adsorbents. This report gives an overview of how polyacrylonitrile has been functionalized using different techniques to formulate effective adsorbents that were then successfully used for wastewater treatment.

## 2. Heavy Metals and Their Effects

**Figure 2:**
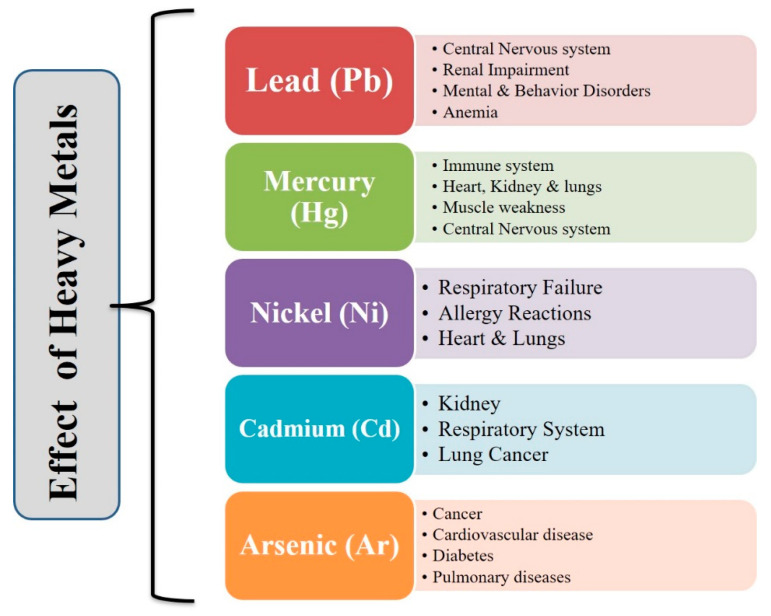
Effects of heavy metals on the health of living systems.

Heavy metals enter water bodies through leaching from rocks, soils, and various human activities such as mining, use of pesticides, insecticides, paints, and power plants [24]. Generally, heavy metals are high atomic weight elements with a high density (more than water). Lead (Pb), mercury (Hg), arsenic (As), chromium (Cr), cadmium (Cd), and nickel (Ni) are examples of heavy metals. These toxic metals are metabolic toxins and compound inhibitors [3]. However, metal ions such as copper, molybdenum, manganese, zinc, iron, boron, and nickel are vital for plant growth. These metals behave as micronutrients for animals and humans as well if they are present in permissible amounts [2]. The tolerable upper intake levels for humans of copper, molybdenum, manganese, zinc, iron, boron, and nickel are 10 mg/day, 2 mg/day, 11 mg/day, 40 mg/day, 45 mg/day, 13 mg/day, and 1 mg/day, respectively. Heavy metals play an important role in many biological and chemical reactions in living organisms and act as enzymes in many redox reactions. However, they are not biodegradable and tend to get accumulated in living beings, causing different ailments and disorders, as depicted in Figure 2. They may also cause mental retardation and brain damage. These metals are exceptionally lethal and can cause harmful impacts even at low concentrations [3]. Heavy metals may enter the living system by means of inhalation, ingestion, and skin penetration. Progressive accumulation of poisonous metals may cause serious health issues like fatigue, pain, neurological disorders, depression, and allergic hypersensitivity.

**Figure 3:**
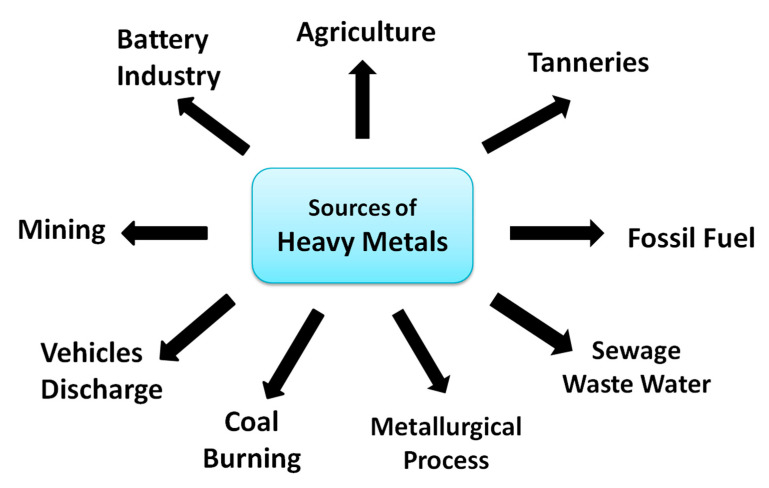
Sources of heavy metal contamination.

Heavy metals are discharged into the ecosystem in a variety of ways (Figure 3), including coal combustion, sewage wastewater, vehicle discharge, the battery industry, mining exercises, tanneries, and the use of fossil fuels [4]. Heavy metals are also utilized as a part of the agriculture industry. Although their wide application has helped in bringing advancement in the economy, heavy metal compounds are dangerous due to their tendency to accumulate in the food chain and in soft (e.g., kidney) and hard tissues (e.g., bone). The World Health Organization (WHO) and the Environmental Protection Agency (EPA) have laid down certain permissible limits for the discharge of heavy metals into the environment to reduce water pollution [2].

It is vital to understand, evaluate, and control the toxic metal waste in the environment. Few heavy metals are discussed below:

### 2.1. Cadmium

Cadmium plays no role in the functioning of the human body and can cause toxic effects even in trace amounts [25]. Cadmium has been produced as a byproduct of zinc and lead refineries, electroplating industries, paints, nickel–cadmium battery industries, textiles, pesticides and insecticides, solders, television, etc. It is also used as a neutron absorber in nuclear reactors. Cadmium is highly soluble in water, so it can easily enter plants and aquatic life forms and is harmful to organisms, even in trace amounts. Inhalation through cigarette smoke and intake from food are the main causes of exposure to cadmium; skin absorption occurs rarely. The diseases caused in human beings by exposure to cadmium are kidney disorders, bone deterioration, liver damage, and cancer. The permissible limit of cadmium in water as recommended by the EPA is 0.005 mg/L [24].

### 2.2. Lead

Lead metal is present in the environment due to contaminated water, food, household waste, and industrial waste. Lead is hazardous to biotic species and can stay in the environment for a longer duration [25]. Organolead compounds such as tetraethyl lead have distinct toxic effects compared with inorganic forms (e.g., lead oxide, lead chromate). The main sources of lead are paints, petroleum products, lead acid batteries, vehicle industries, electroplating industries, etc. Lead is more toxic in its ionic form, such as Pb^2+^/Pb^4+^, because it can easily substitute calcium, magnesium, iron, and sodium cations in cells and disrupt biochemical reactions. A higher concentration of lead in plants is responsible for the destruction of their lipid membranes due to the formation of reactive oxygen species (ROS) which finally disturb the photochemical processes and inhibit the development of the plant. The permissible limit of lead in water as recommended by the EPA is 0.015 mg/L [24].

### 2.3. Mercury

Mercury is extremely poisonous and easily accumulates in living organisms. In nature, it exists in three forms: elemental Hg(0), inorganic compounds (HgCl_2_, HgS, HgO), and organic molecules; each form has a different toxicity, use, and characteristics. The inorganic form of mercury, which is more soluble and reactive towards chemicals, results in the easy deposition of mercury. Many organic compounds such as methylmercury, ethylmercury, and dimethylmercury are found in nature, but methylmercury is the most common form of mercury [26]. Mercury is used in battery industries, semiconductors, chemical industries, dental amalgam, and the agriculture industry. Mercury acts as a neurotoxin and tends to retard mental operations like cognizant, auditory, and sensory functions. Mercury has adverse effects on the kidneys and muscles, causes deformation of the structure of proteins, disorders of the central nervous system, and respiratory problems. The permissible limit of mercury in water as recommended by the EPA is 0.002 mg/L [24].

### 2.4. Arsenic

It is found in literature that many human beings are affected by arsenic exposure, predominantly in countries such as Bangladesh, India, Chile, Uruguay, Mexico, and Taiwan, where underground water is polluted with large amounts of arsenic. In water, its permissible limit is 10 ppb, even though its higher concentration is found in mineral resources. Arsenic is a metalloid present in a trace amount in nature. It is found primarily in organic forms such as monomethylarsonic acid, dimethylarsinic acid, and trimethylarsine oxide, as well as inorganic forms such as arsenite As^3+^ and arsenate As^5+^. In general, inorganic arsenic is more hazardous in comparison with organic forms. Moreover, arsenite is more poisonous as it is more soluble in water than arsenate. The sources of arsenic are volcanic explosions, abrasion of soil, agriculture, industry, dyes, electronic industry, and medical fields. Arsenite binds to the thiol moiety on proteins and enzymes during biochemical reactions and deactivates around 200 enzymes. It can cause neurologic problems, lower RBC counts, resulting in anemia, and lower white blood cell counts, resulting in leucopenia. Exposure to high concentrations of arsenic causes vomiting, diarrhea, and pulmonic hypoxemia. The permissible limit of arsenic in water as recommended by the EPA is 0.01 mg/L [24].

### 2.5. Nickel

The heavy metal nickel has a silver-gray color and can be found in oxidation states +2, +3, and +4. It is employed in the production of various alloys, including nichrome, monel, permalloy, stainless steel, nickel silver, and others, for use in a variety of applications, including high-temperature resistance, corrosion-resistant materials, alnico used for permanent magnets, coatings, desalination plants, household appliances, storage batteries, chemical and food processing equipment, and as a catalyst for hydrogenation in coinage. During nickel mining and in industries that manufacture or use nickel, nickel alloys, or nickel compounds, nickel is emitted into the atmosphere. These industries can also release nickel into effluent [27]. Oil-burning power stations, coal-burning power plants, and garbage incinerators are further sources of nickel emissions into the atmosphere. Depending on the dose and duration of exposure, nickel can harm the immune system and cause cancer and other health problems, such as an itchy rash caused by direct contact with a substance, heart disease, asthma, lung fibrosis, and cancers of the lung and nasal sinus. More significantly, the optimum nickel concentration in drinking water should be kept at 0.01 mg/L, and in industrial effluents, it should be between 0.2 to 2.0 mg/ L [28]. 

### 2.6. Dyes

Dyes are substances that give color to objects such as textiles, paper, and leather. Dyes are introduced into water bodies and the environment through industries such as food, textiles, cosmetics, pharmaceuticals, etc. The dyes contain toxic metals including lead, cadmium, chromium, zinc, copper, and nickel [29]. Dyes are categorized as cationic, anionic, and non-ionic dyes. A variety of dyes are utilized in different industries, such as acid dyes, reactive dyes, basic dyes, azo dyes, direct dyes, vat dyes, and dispersed dyes [15].

Dyes are naturally and chemically synthesized compounds applied to substances that we want to make colorful. There are various types of dyes containing different chemical substances and are used for different purposes, such as acid dyes for coloring animal fibers, basic dyes used for paper, pigment dyes used in paints, and direct dyes for cotton silk [30]. Natural dyes are derived from plants; these are limited in number and fade fast. Synthetic dyes are found in different varieties, and these cannot be destroyed easily. Dyes present in wastewater discharged from dye manufacturing units are dangerous to biotic beings and the environment. Synthetic dyes can be degraded at high temperature conditions by use of oxidants [31]. Pollution due to dyes contains hazardous chemicals resulting in an increase in various diseases. When dyes are not treated and enter the aquatic system, they cause allergic dermatoses, respiratory diseases, carcinogenesis, and mutagenesis.

## 3. Types of Adsorbents Made from Polyacrylonitrile

The adsorption process is an inexpensive method having a very low operating cost. Furthermore, it causes less toxicity during the extraction process of hazardous metals compared with other conventional methods such as filtration, sedimentation, coagulation, chemical precipitation, solvent extraction, neutralization, etc. The adsorption process involves the transport of substances from the liquid phase to the solid surface. The forces which bind the substances to the solid surface can be physical forces or chemical forces. Based on attractive forces, the adsorption process can take place in two ways: physical adsorption and chemical adsorption. In physical adsorption, also known as physisorption, the attractive forces are Vander Waals forces, due to which adsorption is *multilayered*. A physisorption phenomenon involves weak forces that are reversible and nonspecific in nature, and desorption of solute molecules takes place easily, whereas in chemisorption, the attractive forces are chemical bonds, which result in a monolayered adsorption process. Chemisorption is irreversible, specific in nature, and has a high heat of adsorption due to the chemical bonds involved. Although they are different processes, they work together during the adsorption process. The solute adhered to the solid surface during the adsorption process is called an adsorbate, while the substance on which the solute is sustained is known as an adsorbent [16].

Various adsorbents used for the extraction of toxic metal ions and dyes from wastewater are discussed below.

### 3.1. Polyacrylonitrile in Ion Exchange Resins

Ion exchange resins are high-molecular-weight crosslinked polymers used to exchange the ions present in the wastewater with the ions present in the resin. Ion exchange resins are insoluble in water as well as in organic solutions, and these crosslinked polymers are connected through covalent bonds [32]. Ion exchange resins are of two types, namely, cation exchangers and anion exchangers. There are several research papers available on ion exchange resins that are used to extract toxic ions and dyes. In an initial experiment, the anion exchange resin was obtained by the reaction of highly permeable acrylonitrile–DVB copolymers with hydrazines, ethylene diamine, diethylene triamine, and triethylene tetramine, and their complexing properties towards copper and cobalt ions were explored [33]. The complexation values were found to be 13–53 mg/g for copper and 27–60 mg/g for cobalt. In another study, a redox technique was used to prepare poly(acrylonitrile-co-acrylamide) poly(AN-co- AM) by utilizing sodium bisulfite and potassium persulfate as the initiators [34]. The resultant copolymers were then modified by chemical treatment with hydroxylamine hydrochloride to introduce amidoxime functional moieties into the polyacrylonitrile (PAN) framework. The function of the amidoxime moiety was to develop a chelating ion-exchange resin formed through acrylonitrile for the extraction of toxic metal waste in water. Inductively coupled plasma was used to investigate the sorption behavior of poly(AN-co-AM) for Cu^2+^ ions. The adsorption capacity of Cu^2+^ increased with an increase in pH until optimum adsorption of Cu^2+^ (52.63 mg/g) at pH 5. The adsorption of Cu^2+^ onto poly(AN-*co*-AM) correlates well with the Langmuir equation. A kinetic study showed that the adsorption of Cu^2+^ follows the pseudo-second-order model. The adsorption capacity of Cu^2+^ onto amidoximed poly(AN-co-AM) decreased as the adsorbent amount increased. Zhang et al. (1994) reported the -COOH moiety containing hydrazine immobilized polyacrylonitrile fibers [35]. The highest uptake values of functionalized polyacrylonitrile fiber for the heavy metal ions Cu^2+^, Cd^2+^, Zn^2+^, Co^2+^, Pb^2+^, Cr^3+^, Ni^2+^_,_ and Hg^2+^ were found to be 1.33 mmol/g, 1.30 mmol/g, 1.03 mmol/g, 1.02 mmol/g, 0.98 mmol/g, 0.96 mmol/g, 0.95 mmol/g, and 0.63 mmol/g, respectively. This cation exchanger was selective for different metal ions and was arranged according to their distribution coefficient values as follows: Cu^2+^> Zn^2+^> Cr^3+^> Hg^2+^> Cd^2+^> Pb^2+^> Ni^2+^> Co^2+^. The product could be regenerated and reused after recovery with diluted nitric acid.

A polyacrylonitrile-based adsorbent was synthesized for the separation of cesium and cobalt ions from radioactive waste [36]. Inorganic–organic adsorbent was formed by inorganic ion exchangers modified by polyacrylonitrile having larger size particles with higher granular strength. Potassium nickel hexacyanoferrate (KCNFPAN) was used as an inorganic ion exchanger and polyacrylonitrile as a binding matrix to produce a composite adsorbent. Three different complexing agents based on granular hexacyanoferrate were utilized to extract Cs^+^ and Co^2+^ ions from aqueous solution. The ion exchange ability of the adsorbent was also dependent on the drying temperature of samples; the sorption value of adsorbent KCNFPAN was increased as the temperature reached 110 °C. The highest adsorption values were obtained to be 2.85 mmol/g and 2.15 mmol/g for cesium and cobalt ions, respectively. Shunkevich et al. (2005) synthesized polyacrylonitrile fiber by using various strong and weak base moieties to produce an anion exchange fiber [37]. Potentiometric titration curves were used to study the acid–base characteristics of the anion exchangers by calculating the acidity variables. These ion exchange fibers contained at least four different functional groups and their ion exchange capacity was around 3.5 meq/g. The ion exchange value of resin containing strong base quaternary amino groups (pK≅ 2) was 2.5 meq/g. The nitrile groups in the polyacrylonitrile underwent hydrolysis and introduced carboxylic acid moieties in the ion exchange fibers. Moon et al. (2004) developed ion exchanger beads for the extraction of Sr^2+^ and Ag^+^ ions in acidic conditions [38]. Potassium titanate (K_2_Ti_4_O_9_) and nickelferrocyanate (Ni_2_Fe(CN)_6_) powders were utilized to form inorganic ion exchanger beads. These powders were dissolved in the solvent dimethylsulfuoxide with a small amount of TWEEN-80 surfactant, mixed at 50°C. The polyacrylonitrile powder was then mixed in a known quantity of the above homogeneous solution at a temperature of 323 K to obtain a uniform solution. The diffused air in the solution was removed and a composite solution was passed through a dual nozzle to obtain sphere-shaped beads. It was found that nickelferrocyanate ion exchangers were more selective for Ag^+^ ions in comparison with potassium titanate, while potassium titanate ion exchangers showed more affinity for Sr^2+^ ions than Ag^+^ ions.

Chelating resin belongs to the category of ion exchange resins and contains functional groups connected to the backbone of the main chain polymer. It has one or more electron donor atoms, such as nitrogen, sulfur, oxygen, and phosphorous, that can form coordinate bonds with most of the poisonous heavy metals. A chelating resin containing amidoxime groups was utilized to extract copper ions [15]. This resin was synthesized by the aqueous suspension copolymerization of acrylonitrile and divinyl benzene and subsequent modification of cyano groups by reaction with hydroxyl amine. Another poly(acrylamidoxime) chelate resin was reported by Kim, Shin, and Maeng, (1982), synthesized through redox polymerization of acrylonitrile and divinyl benzene and then reacted with hydroxylamine hydrochloride to generate a functional moiety [39]. Poly(acrylamidoxime) resin’s complex formation properties were discovered to be viable for the extraction of lanthanum, cerium, and neodymium from wastewater. Polyacrylonitrile copolymers containing cysteine groups were synthesized by reacting polyacrylonitrile copolymers with L-cysteine and 1,6 hexanediol. This resin was much more selective towards Ag^+^, Hg^2+^, Au^3+^ and Pt^4+^ in diluted acidic conditions; the highest adsorption values were found to be 0.97 millimole, 0.65 millimole, 1.22 millimole, and 0.39 millimole per gram of resin [40]. 

In another experiment, substituted polyamides were formed by reacting acrylonitrile–vinylacetate–divinylbenzene copolymer with amino guanidine bicarbonate for effective removal of gold cyanide (49.3 mg/g) in a butanol–water mixture [41].

Various resins with guanidyl groups are also reported. Oswik et al. reported the adsorption of copper by the resin containing guanidine ligand derivatives [42]. The aminolysis of poly(acrylonitrile–vinylacetate–DVB) was carried out by utilizing ethylene–diamine or hydroxyl amine hydrochloride after their reaction with dicyandiamide, cyanamide, and sodium dicyanimide. The resultant resins had biguanidyl, guanidyl, or nitrilguanidyl derivatives in the polymer side chain, respectively. The structure of ligand complexes with copper ions was determined through electron paramagnetic resonance spectroscopy. Arsalani and Hossein Zadeh (2005) have reported ethylenediamine containing polyacrylonitrile for the extraction of toxic ions [43]. The highest and lowest metal adsorption values were 5.2 millimole per gram for Ni^2+^ at pH 5.0 and 1.5 millimole per gram for Co^2+^ at pH 1.0, respectively. Jermakowicz–Bartkowiak, Kolarz, and Serwin (2005) investigated functionalized copolymers by reacting poly (vinyl benzyl chloride–acrylonitrile–divinyl benzene) matrix with ethylene diamine, bis(aminopropyl) amine, bis(aminohexyl) triamine, and tris(2-amino ethyl) amine followed by reaction with 5-ethylthiourea to obtain a copolymer having 2.8–5.0 millimole of amino moiety per gram of resin [44]. The adsorption value of the above copolymer for gold, platinum, and palladium chloro complex were determined in 0.1 M HCl acidic solution and were found to be 190 mg/g, 245 mg/g, and 280 mg/g of resin, respectively. Another chelating resin, polyacrylonitrile-2-amino-1,3,4-thiadiazole (PAN-ATD) was developed through a single step reaction and its composition was described through FT-IR [45]. The adsorption of mercury ions by the resultant copolymer was examined through batch and column techniques. The results obtained through the batch method demonstrated that polyacrylonitrile-2-amino-1,3,4-thiadiazole had an affinity for mercury ions and the highest adsorption value assessed from the Langmuir method was 526.9 mg/g at 308 K. Moreover, this resin was more selective for mercury ions in the mixture of metal ions such as Hg^2+^, Ni^2+^, Cu^2+^, Cd^2+^, Pb^2+^, and Zn^2+^ at the same pH.

Morcali and Zeytuncu (2015) synthesized thiourea- and glutaraldehyde-modified polyacrylonitrile adsorbents [46]. The parameters affecting the adsorption process, such as pH range (0.70 to 2.00) and adsorbent dose (100 to 800 mg), were studied for different time intervals. The optimum pH for the extraction of platinum and palladium was found to be 1.5. The Langmuir isotherm model illustrates the adsorption of toxic ions by the adsorbent much more precisely compared with the Freundlich model. It was found that the extraction rate for platinum and palladium reached around 93% in 0.50 M HCl–0.50 M thiourea solution and no noteworthy decline after 5 adsorption–desorption cycles was obtained.

The adsorption of Cu^2+^ and Cr^2+^ on pumice and a polyacrylonitrile/pumice composite was examined by Yavuz et al. (2008) by utilizing a batch technique at room temperature, and their adsorption properties were evaluated on pumice and the composite [47]. The sorbent’s adsorption rates were discovered to vary with their properties, such as surface functionalization, preliminary concentration of metal ions solution, adsorption kinetics, and solution pH. The time needed to remove metal ions from wastewater was six hours, and solution concentrations were in the range of 100–500 mg/g of resin. Langmuir and Freundlich isotherm models were utilized to determine the amount of adsorption of Cu^2+^ and Cr^3+^ on the adsorbent and results were found to fit in the Langmuir models. The Langmuir and Freundlich isotherm models were utilized to determine the amount of adsorption of Cu^2+^ and Cr^3+^ on the adsorbent, and the results were found to fit in the Langmuir models. The extraction of Cu^2+^ and Cr^3+^ from the metal ion mixture was greater in the composite than in pumice. The adsorption values of the sorbents pumice and polyacrylonitrile/pumice composite observed from the isotherm equations were 0.055 and 0.065 for Cu^2+^ and 0.031 and 0.268 mmol/g for Cr^3+^ ions, respectively. In another study, the amidoximated chitosan-grafted polyacrylonitrile (CTS-g-PAO) was reported to adsorb UO_2_^+^ from aqueous solution [48]. The adsorption capacity of CTS-g-PAO was found to be 312.06 mg/g and equilibrium was attained within 120 min. Maximum loaded uranium on CTS-g-PAO (more than 80%) was desorbed through 0.1 M HCl or EDTA-Na, CTS-g-PAO, and reutilized for at least three cycles.

Y. Park et al. (2010) examined heavy metals such as cobalt, strontium, and cesium released into water bodies through radioactive industries and removed them by using an ammonium molybdophosphate–polyacrylonitrile (AMP–PAN) adsorbent [49]. In a single and binary solution of metal ions, the competitive adsorption of Co^2+^, Sr^2+^, and Cs^+^ by ammonium AMP-PAN was examined. The binary system of metal ions Sr^2+^/Co^2+^, Sr^2+^/Cs^+^, and Co^2+^/Cs^+^ were synthesized by combining the similar metal concentration solution with an identical volume ratio for every metal. Binary metal adsorption performance was best explained by the Langmuir model. The Freundlich, Langmuir, and D–R models were best fitted to the adsorption records by using the non-linear regression system. The adsorption model demonstrated that AMP–PAN was more selective for Cs^+^ ions and the maximal uptake values were 0.16 mmol/g, 0.18 mmol/g, and 0.61 mmol/g for Co^2+^, Sr^2+^, and Cs^+^, respectively. Due to competition among metal ions in a binary system, adsorption of one metal ion was suppressed in the presence of another metal ion. Alkali metals (Na^+^) inhibited the adsorption of cesium and calcium ions and diminished the adsorption of cobalt ions onto AMP–PAN. Adsorptions of Co^2+^, Sr^2+^, and Cs^+^ onto AMP–PAN in the vicinity of surfactants were quite dissimilar. The presence of cationic (OTMA and HDTMA) and anionic (SDBS and SOBS) surfactants diminished the adsorption of Co^2+^, Sr^2+^, and Cs^+^ onto AMP–PAN. However, the presence of non-ionic surfactants (Tween 80 and Triton X-100) did not decrease the adsorption. 

The zirconium–manganese oxide/polyacrylonitrile (Zr–Mn oxide/PAN) composite was synthesized in another study and used to remove Sr^2+^ ions from wastewater [50]. Here, zirconium–manganese hydrous oxide was used as an inorganic active ion exchanger to prepare composite spheres. Zr–Mn mixed oxide powder was obtained through the co-precipitation method and then blended with polyacrylonitrile (PAN) to produce Zr–Mn oxide/PAN composite spheres. The adsorption process was carried out with an initial metal ion concentration of 20 ppm at pH 7.7 and a temperature of 324.2 K. The adsorption of Sr^2+^metal ions was investigated at concentrations ranging from 20 to 200 ppm, and the results were explained using Freundlich, Langmuir, and D–R models. Hydrous manganese dioxide–polyacrylonitrile (MnO_2_–PAN) spheres were chemically prepared and used for the removal of radio-contaminant cesium-137 from metal ion solutions [51]. Inductively coupled plasma was used to study manganese and other elements such as carbon, hydrogen, and nitrogen, determined through the CHN elemental analyzer. MnO_2_–PAN adsorbent showed thermal stability up to 583 K and had a crystalline structure. Zong et al. (2011) developed a novel method for the modification of the surface of *N*-chlorosulfonamidated polystyrene through activators recovered by electron transfer and atom transfer radical polymerization (ARGET ATRP) methods [52], achieved by the use of ferric trichloride anhydrous (FeCl_3_)/iminodiacetic acid (IDA) as a catalyst, L-ascorbic acid (VC) used as a reductant, and *N*,*N*-dimethylformamide (DMF) as the solvent. Polyacrylonitrile was bound to the surface of polystyrene and the cyano moiety of the PAN-g-PS polymer was treated with hydroxylamine hydrochloride to introduce amidoxime (AO) groups in the PAN chain. The amidoxime moieties were efficient for the extraction of Hg^2+ ^ions from solutions. Adsorption phenomena were also investigated in binary metal systems such as Hg^2+^-Pb^2+^, Hg^2+^-Ag^+^, and Hg^2+^ -Cu^2+^, with the results indicating that resin exhibited selectivity for mercury ions. A biosorbent was produced by immobilizing dead fungal biomass on polyacrylonitrile to examine the ability of microorganisms to extract metal ions from solution [53]. This adsorbent was used for the extraction of hazardous metals such as Cu^2+^, Zn^2+^, and Ni^2+ ^from the mixtures. The metal removal capacities of the adsorbent (concentrations 50%) were 16 mg/g, 7 mg/g, and 0.25 mg/g at pH 7 for Zn^2+^, Cu^2+^, and Ni^2+^, respectively. The adsorption values of PAN (concentrations of 75%) were 18, 7.9 mg/g and 0.25 mg/g for Zn^2+^, Cu^2+^, and Ni^2+ ^ions, respectively. Dong, Chen, and Gao (2007) used an γ-ray irradiation technique to connect a partially deacetylated chitin (deacetylation degree of around 40%) to acrylonitrile. The maximum binding value of acrylonitrile onto the pre-irradiated chitin at 25 ± 1.2 kGy was 114% for an acrylonitrile concentration of 40% (*v*/*v*) in dimethylformamide at 343 K for eight hours. The mixture ratio of 0.1 N hydroxylamine hydrochloride to 0.1 N sodium hydroxide was chosen to be 7:3 (*v*/*v*) for the conversion of cyano groups on grafted chitin (Chi-g-AN) into amidoxime. The equilibrium value of As^3+^ on Chi-g-AN converted amidoxime (Chi-g-AN-C) was well fitted in the Langmuir model and its highest adsorption value was 19.724 mg/g.

Different polymeric materials were reported by Kiani et al. for the extraction of Ag^+^ and Pb^2+^ ions from synthetic solutions [54]. They treated the polyacrylonitrile with triethylenetetramine (PAN–TETA 50 and 100) using different amounts. Polyacrylonitrile–triethylenetetramine and their metal complexes were investigated through FT-IR and SEM. At pH 6.0, the highest uptake values for Ag^+^ and Pb^2+^ ions were 108.14 mg/g and 99.01 mg/g, respectively. Desorption of Ag^+ ^and Pb^2+^ ions were done in 0.5 M HCl acidic solution and the desorption value was found to be 91%. In another study, a permeable acrylonitrile (AN)/methyl acrylate (MA) copolymer was prepared through the suspension emulsion polymerization method and the resultant copolymer was functionalized with hydroxylamine hydrochloride to form an amidoxime group to extract toxic ions from wastewater [55]. The results confirmed that amidoximated AN/MA beads showed good adsorption values for Hg^2+^, Ag^+^, and Cu^2+^ with great selectivity for Hg^2+ ^ions. The equilibrium adsorption values of amidoximated AN/MA copolymer for various ions were found to be 2.68 mmol/g for mercury ions, 1.35 mmol/g for silver ions, 2.01 mmol/g for copper ions, 0.74 mmol/g for iron ions, and 0.06 mmol/g for lead ions, with the initial concentration of metal ions solutions being 5.0 mM. On the other hand, untreated AN/MA did not show any removal efficiency. The equilibrium was attained within 10 h through adsorption kinetics. Another study reports the development of crosslinked acrylic acid (AA)-acrylonitrile (AN) resin through the suspension polymerization method by using poly(vinyl alcohol) as a suspending agent and *N*,*N*-methylenebisacrylamide (MBA) and divinylbenzene (DVB) as crosslinkers [56]. Acrylonitrile and acrylic acid were used in a mole percent ratio of 95:5, while various ratios of crosslinking agents (2.0, 5.0, and 10.0 weight percent) were used during the formation of the copolymer. The adsorption of uranium took place in HNO_3_, HCl, and H_2_SO_4_ solutions. This paper also discusses the impact of different parameters such as pH, time to adsorb toxic ions, kind of acid, nature of crosslinking agents, their amounts, and desorption phenomena. In another study, the synthesis of iminodiacetate functionalized acrylonitrile–divinylbenzene (AN-DVB) copolymers by using crosslinkers at amounts of 10 and 15 weight percent is reported. These chelating resins were utilized to extract Pb^2+^, Cd^2+^, and Zn^2+^ from wastewater through batch and column methods. The amount of metal ions adsorbed by resin was studied through Freundlich and Langmuir isotherm models. It was found from the results that chelating resins adsorbed the ions in single metal systems in the order Cd^2+^ > Pb^2+^ > Zn^2+^. Monodispersed poly(acrylonitrile) was synthesized through a surfactant-free emulsion polymerization technique and the nitrile moiety was transformed into amidoxime on reaction with hydroxylamine hydrochloride in water [57]. The synthesized resin was used as an adsorbent in the extraction of three types of contaminants; methylene blue (MB) and rhodamine 6G (R6G), a heavy metal ion Cd^2+^, and the herbicide paraquat. The various factors that influenced the adsorption rate were amidoxime concentration, pH of contaminated water, adsorbent dose, and initial metal ion concentration. Amidoximated poly(acrylonitrile) polymer extraction ability was increased to 87 mg/g, 91 mg/g, 74 mg/g, and 91 mg/g from 5 mg/g, 1.54 mg/g, 1.06 mg/g, and 1.22 mg/g for Cd^2+^, methylene blue, rhodamine 6G, and herbicide paraquat, respectively. D. J. Wang et al. (2014) reported polyacrylonitrile embedded on the surface of a wheat straw matrix (WSM) via single-electron transfer-living radical polymerization (SET-LRP) [58]. Wheat straw matrix grafted polyacrylonitrile was converted into wheat straw matrix grafted-amidoxime group by reacting with hydroxylamine hydrochloride. Wheat straw matrix grafted-amidoxime was used to extract mercury metal ions with the utmost adsorption ability of 4.7 mmol/g. It was found that in the binary system of metal ions Hg^2+^- Pb^2+^, Hg^2+^- Ni^2+^, Hg^2+^-Zn^2+^, Hg^2+^- Ag^+^, and Hg^2+^- Cu^2+^, the above adsorbent showed high selectivity for mercury ions. As per the acid–base theory, mercury ions form stable complexes with the ligands containing nitrogen and sulfur atoms. Feng et al. explained the preparation of polyacrylonitrile containing carbon materials (PPC-0.6-600, PPC-0.8-600, PPC-0.6-800, and PPC-0.8-800) [59]. These materials were synthesized by heating carbon with potassium hydroxide at 600 °C and 800 °C for the extraction of chromium ions from aquatic systems. The adsorption value of chromium ion on PPC-0.8-800 was more than other carbon materials, and the greatest adsorption capacity was found to be 374.90 mg/g at pH 2.0. This resin could be reused even after five cycles. This adsorption process was due to the electrostatic force between polyacrylonitrile-based porous carbon materials and hydrogen chromate (HCrO_4_^−1^) ions. For the extraction of Pb^2+^ ions, an ethyleneamine functionalized crosslinked poly(acrylonitrile-ethylene glycoldimethacrylate) chelating resin was synthesized [60]. This resin was modified with different amine groups such as ethylenediamine, triethylenetetraamine, and tetraethylenepentamine. The maximum extraction value for toxic metal lead ions was obtained at pH 4.6 in the pH range of 1–4.6 studied for all copolymers. The adsorption of Pb^2+^ ions was conducted with an artificial metal ion solution having a concentration of 0.483–2.4154 mmol/L at pH 4.6. Maximum uptake values of lead ions were 1.73 mmol/g, 1.68 mmol/g, and 1.62 mmol/g, respectively. In another experiment, an adsorbent was prepared by graft polymerization of polyacrylonitrile with arabic gum (PAN–g–AG) by using potassium permanganate or nitric acid as a redox initiator for the extraction of lead, cadmium, and copper ions from their solutions [61]. The grafted copolymer was modified with hydrazine and then hydrolyzed with sodium hydroxide. The batch adsorption method was used for the extraction of the metal ions from a solution having an initial concentration of 100 ppm by using 50 mg of improved PAN–g–AG for four hours. Results showed that maximum adsorption of lead ions took place at pH = 5, while pH = 6 was suitable for cadmium and copper ions. The comparison between PAN–g–AG and polyacrylonitrile fibers indicated that PAN–g–AG had much better adsorption capacity for toxic metal ions compared with polyacrylonitrile fibers. Malachite green (MG) dye is utilized in cloth mills, leather manufacturing units, paper industries, and as a fungicide, fish cannery, and antibacterial agent. Its exposure to living beings has a toxic effect on various organs such as the liver, kidney, lung, spleen, and skin. Therefore, an adsorbent of thiourea functionalized poly (acrylonitrile–acrylic acid) was prepared to remove dye MG [62]. This copolymer was synthesized by acrylonitrile (97.0%) and acrylic acid (3.0%) through the redox polymerization method at 60°C by using sodium bisulfate and potassium persulfate as initiators. It was then reacted with thiourea to produce a thiourea functionalized copolymer. A solution of malachite green having a different concentration (20 to 100 ppm) was used. Adsorption equilibrium was obtained with a 100 mL solution of a concentration of 20 to 100 ppm by using 0.002 g of adsorbent dosage. Regeneration of the adsorbent was done through a mixture of 1.0 mol/L of nitric acid and 0.5 mol/L of thiourea by shaking the solvent with the adsorbent for three hours. New sulfonated poly(acrylonitrile-co-styrene) and poly(acrylonitrile-co-styrene) nanocomposites were made using a precipitation polymerization technique, and multiwall carbon nanotubes were synthesized using chemical vapor deposition. To remove the methyl orange dye from the aqueous solution, sulfonated and multiwall carbon nanotubes were impregnated into the poly(AN-co-St) nanocomposite at a 1:1 ratio [63]. The Langmuir isotherm determined that the maximal adsorption values for poly(AN-co-St)/MWCNTs, sulfonated (AN-co-St), and poly(AN-co-St) composites were 121.95 mg/g, 48.78 mg/g, and 47.84 mg/g, respectively. Furthermore, when compared with previous studies, the maximal sorption capacity of poly(AN-co-St)/MWCNTs was 121.95 mg/g. Particle size distribution and scanning electron microscopy images were used to show the morphology of the nanocomposite molecules.

### 3.2. Polyacrylonitrile as Fiber 

**Figure 4:**
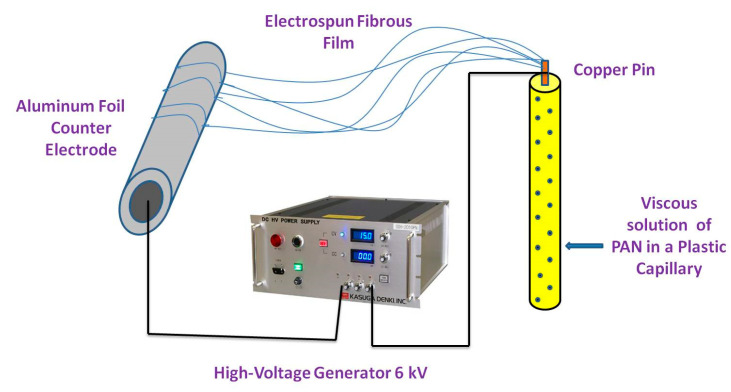
Schematic diagram for preparation of nanofibers through the electrospinning method.

Polyacrylonitrile fiber has many favorable characteristics, such as elasticity, huge surface area, and excellent toughness, making it a super adsorbent [64]. The polyacrylonitrile structure contains semi-crystalline and amorphous phases. Polyacrylonitrile contains a large number of carbon–carbon in its formula, which is responsible for its stability and excellent toughness. It is used to produce carbon nanofibers owing to its high carbon content (56%), and thus, stable products are formed through the reaction of the nitrile group [65]. Many research papers have discussed the techniques for forming nanofibers such as vapor growth, arc discharge, laser ablation, and chemical vapor deposition. However, these methods are very costly and the product yield is very poor. Electrospinning is a new method employed to produce fibers from polymer solutions under electrostatic forces. The components used in the electrospinning process are the high-voltage power supply, syringe pump, spinneret, and collector (Figure 4). In this process, high voltage is applied to a polymer viscous solution obtained by dissolving polymer into a suitable solvent and spinning it by applying high voltage current.

Many researchers have reported different modified fiber adsorbents of polyacrylonitrile, as discussed below.

Shubo Deng and Bai (2004) utilized aminated polyacrylonitrile fibers (APANFs) as adsorbents for the evacuation of Cr^3+^ and Cr^6+^ species from wastewater at various values of pH [66]. The adsorption values demonstrated that large quantities of Cr^3+^ and Cr^6+^ species are absorbed using APANFs, even though the adsorption values depend on the pH. As per the results, the amount of adsorbed of Cr^3+ ^ions increased with the increase in pH in the range of 1.5 to 6.5, and adsorption of Cr^6+ ^was decreased with the rise in pH of the solution in the range of 2.4 to 12. The adsorption performances of Cr^3+^ and Cr^6+^ on aminated polyacrylonitrile fibers varied according to the forces between metal and adsorbent. A kinetic study revealed that adsorption equilibrium was obtained within one to two hours. Recovery of Cr^6+^ ions from the adsorbent APANFs was done in basic conditions and reutilized without loss of adsorption value, while Cr^3+^ ions recovery in acidic conditions was not very effective. The adsorption of metal on oxime-modified polyacrylonitrile nanofibers was studied by Saeed et al. (2008). They obtained functionalized polyacrylonitrile nanofibers by reacting hydroxylamine hydrochloride with the cyano group of polyacrylonitrile [67]. The maximal adsorption uptake values for Cu^2+^and Pb^2+^ were 52.70 mg/g and 263.45 mg/g, respectively, obtained from the Langmuir adsorption isotherm, indicating that the adsorption was a single layer on nanofiber. The desorption of metal stacked on the above adsorbent could be done by 1.0 M HNO_3 _acid. Acrylonitrile was introduced into polypropylene fiber by γ-rays irradiation followed by reaction with diethylene triamine, resulting in an aminated chelating fiber with a high adsorption value for Hg^2+^ ions [68]. The functionalized chelating fibers were found to be much more suitable adsorbents for mercury ions, and the maximum adsorption value for mercury ions was 657.9 mg/g. The maximum adsorption value of mercury ions was due to attraction between the NH_2_ moiety and Hg^2+ ^ions. Hydrolyzed polyacrylonitrile fibers have also been successful in the extraction of copper ions [69]. Polyacrylonitrile and PAN fibers functionalized with amines were used to remove lead and nickel individually from the aqueous medium by the hexamethylenediamine-modified polyacrylonitrile nanofibers and utilized for the extraction of toxic ions of lead, copper, and nickel from the artificial solution of metal ions [21,54,70,71]. The electrospinning method was utilized to make polyacrylonitrile nanofiber mats, which were then improvised through hexamethylenediamine to obtain the aminated polyacrylonitrile (APAN) nanofiber mats. The obtained nanofiber mat was utilized to extract three kinds of metal ions, namely, Pb^2+^, Cu^2+^, and Ni^2+^ ions from aqueous solution. The maximum uptake values for Pb^2+^, Cu^2+^, and Ni^2+^ metal ions on the APAN nanofiber mats were 175, 162.5, and 48.5 milligrams per gram of adsorbent, respectively. Another study examined the effectiveness of removing Cr^3+^, Pb^2+^, and Hg^2+^ ions from industrial effluents using hydrolyzed/thioamidated polyacrylonitrile fibers [72]. The adsorption values for Cr^3+^, Hg^2+^, and Pb^2+^ were found to be 0.73 mmol/g, 0.09 mmol/g, and 0.14 mmol/g (at pH 4.0), respectively. This adsorbent was very selective for the Cr^3+^ ions compared with Pb^2+^ and Hg^2+^ ions. 

**Figure 5:**
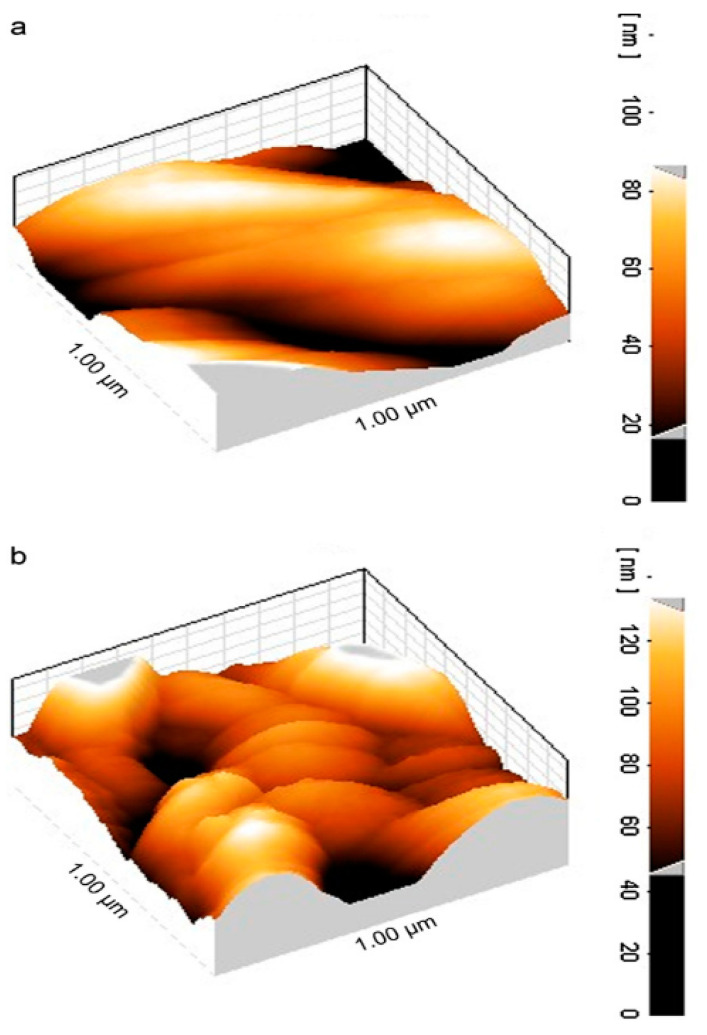
Three-dimensional AFM images of (**a**) aminated PAN-NF before adsorption and (**b**) after copper adsorption. Adapted with permission from reference [73].

Neghlani, Rafizadeh, and Taromi (2011) synthesized polyacrylonitrile nanofibers by the electrospinning method and modified them by diethylenetriamine to form aminated-polyacrylonitrile nanofiber membranes [73]. These membranes were utilized to remove Cu^2+^ ions from synthetic solutions. The percentage of amine loading on fibers was calculated by the gravimetric method. Atomic absorption spectroscopy (AAS) was utilized to determine the concentration of Cu^2+^ ions in the aqueous solution after adsorption. The maximum adsorption value achieved through the Langmuir model for Cu^2+^ ions was 116.522 mg/g. Atomic force microscopy was used to study the changes in morphology before and after the adsorption of copper ions, as shown in Figure 5.

Huang et al., 2013 produced polyacrylonitrile nanofibers through an electrospinning process and then functionalized them with-NH_2_OH to form chelating nanofibers [74]. These nanofibers were used to extract Fe^3+^ and Cu^2+^ ions. Amidoxime-containing polyacrylonitrile nanofibers (AOPAN) showed the highest uptake values of 320 mg/g and 380 mg/g for Cu^2+^ ions and Fe^3+^, respectively. The adsorption value of Fe^3+^ by the adsorbent was found to be higher compared with Cu^2+^ ions. Higher affinity for iron ions towards nanofibers could be explained on the basis of the hard–soft acid base theory. The maximum uptake value was dependent upon attractions between AOPAN and metal ions with similar initial concentrations. Another study reported the synthesis of activated carbons from polyacrylonitrile fibers and investigated their ability to remove toxic ions from wastewater [75]. Polyacrylonitrile fibers were oxidized in the presence of air in a temperature range of 463 K to 553 K for 3–4 days. Activated carbon was produced by treating the fibers at a higher temperature (553–573 K) and keeping them until the fibers converted to hard carbon deposits with the release of smoke. This carbon is then transformed into a powdery form before the activation. 

**Figure 6:**
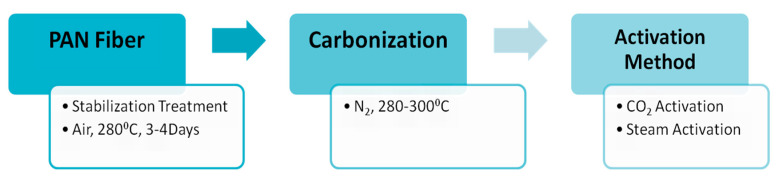
Activated carbon derived from PAN fibers.

*Carbon dioxide* and *steam are* the most common *activating* agents used for polyacrylonitrile oxidizing fibers (Figure 6). Two forms of commercially available activated carbon fibers, A-20 and W10-W, were used for comparable studies. It was found that the steam activation method produced a large surface area but low product of activated coke compared with carbon dioxide.

In another study, the synthesis of polyacrylonitrile–polyamidoamine (PAMAM) composite nanofibers was done by an electrospinning scheme to investigate their dye removal ability [76]. Direct red 80 (DR80) and direct red 23 (DR23) with concentrations of 40 ppm were utilized to study the adsorption capacity of the resultant fibers. The factors such as adsorbent dose, initial dye concentration, and pH of the solution affected the dye removal process. Pseudo second-order kinetics and Langmuir isotherm were used to study the time taken to extract metal ions and the concentration of metal ions to be adsorbed. The maximum uptake value of dyes by PAN/PAMAM composite nanofibers was 1666.66 mg/g and 2000 mg/g for DR80 and DR23, respectively.

The preparation of polyacrylonitrile/polypyrrole (PAN/PPy) core–shell structure nanofibers was carried out through an in situ chemical oxidative polymerization technique to extract Cr^6+^ from wastewater [77]. The pH was found to be an important parameter to affect the adsorption of Cr^6+ ^ions; adsorption of Cr^6+^ ions was enhanced with a decrease in the pH of the solution. The time taken to reach equilibrium was 30 min and 90 min, with the change in initial concentration from 100 ppm to 200 ppm. The results agreed with the pseudo-second-order model. Horzum et al. (2012) contemplated a method to synthesize polyacrylonitrile (PAN) fibers by an electrospinning method for the removal of radioactive uranium particles from aqueous systems via column adsorption under continuous flow [78]. These fibers were functionalized with NH_2_OH.HCl to convert nitrile moieties into amidoxime moieties. Amidoximated polyacrylonitrile fibers could be used repeatedly, however, their adsorption tendency decreased regularly. L. Zhang et al. (2009) investigated functionalized polyacrylonitrile fiber by reacting it with diethylenetriamine and chloroacetic acid to form a reusable chelating fiber containing polyamino–polycarboxylic acid ligands [79]. The immobilization of amine and carboxymethylation into polyacrylonitrile fibers was determined through X-ray diffraction, SEM, and FT-IR. These chelating fibers decreased the Cd^2+^ ions concentration in sewage water by up to 0.001 ppm, although the highest adsorption value of Cd^2+^ ions was 1.34 mmol/g. In addition, the resultant adsorbent was highly efficient for treating other metal ions such as Cu^2+^, Ca^2+^, Zn^2+^, Mg^2+^, Pb^2+^, Ni^2+^, Ag^+^, and Hg^2+ ^ions. Another study reports acrylic fibers synthesized and improved by diethylenetriamine to produce modified chelating fibers [80]. This functionalized resin was utilized to remove Pb^2+^, Cu^2+^, and Ce^4+ ^ions from water. It was found that the mechanical strength of modified resin was low compared with unmodified resin. In other research, activated carbon nanofibers (ACNFs) were synthesized from the precursor polyacrylonitrile and magnesium oxide through an electrospinning process [81]. Adsorption of cadmium ions was investigated using different adsorbents such as polyacrylonitrile-based activated carbon nanofibers/magnesium oxide, pristine, activated carbon nanofibers, and granular-activated carbon. A batch adsorption study showed that polyacrylonitrile-based activated carbon nanofibers/magnesium oxide and activated carbon nanofibers were best in the extraction of cadmium compared with granular activated carbon. The polyacrylonitrile/magnetite nanofibers were prepared via an electrospinning process followed by chemical precipitation by magnetite (Fe_3_O_4_) [82]. It was a two-step process. Iron nanoparticles tend to accumulate and settle down at the bottom, making them less effective in the removal process. Therefore, iron nanoparticles were loaded onto polyacrylonitrile to enhance their efficiency in the extraction of metal ions. A batch method was used for the extraction of lead ions using polyacrylonitrile/magnetite nanofibers. An equilibrium time study was conducted with a 50 mg/L solution for different time intervals. It was observed that maximum adsorption capacity for lead ions was obtained within 80 min. Afterwards, there was no change in the adsorption capacity. The polyacrylonitrile-based calixarene nanofibrous sorbents were synthesized by an electrospinning process for the extraction of Cr^6+^ ions [31]. Calixarene molecules having piperidine groups at the lower or upper ends of their calix structure were used as water-resistant composite nanofiber adsorbents. This adsorbent showed the greatest sorption for chromium ions. Moreover, the thermal stability of calixarene immobilized polyacrylonitrile nanofibers was found to be greater than that of polyacrylonitrile. E. F. Chaúque et al. (2016) synthesized polyacrylonitrile nanofibers through an electrospinning method [83]. The surface of the fibers was modified with poly ethylenediaminetetraacetic acid (EDTA) using ethylenediamine (EDA) as the cross-linker. The resultant fibers were used for the removal of Cd^2+ ^and Cr^4+ ^ions from aqueous solution. The utmost adsorption by modified fibers was 32.68 mg/g and 66.24 mg/g for Cd^2+ ^and Cr^4+ ^ions, respectively, at 298 K. Regeneration of nanofibers could be done through a 2M HCl solution. Another study reports activated carbon nanofibers (ACNFs) developed from precursor polyacrylonitrile and manganese oxide (MnO_2_) through an electrospinning technique [84]. For the comparison of the extraction of Cd^2+^ ions from the aqueous solution, ACNFs, composite ACNFs, and commercial powdered activated carbon were used to make a comparison. From the adsorption data, it was found that Cd^2+^ ion extraction by activated carbon nanofibers/MnO_2 _was maximum (97%), followed by activated carbon nanofibers (96%), and then followed by powdered activated carbon (74%). Silver-coated polyacrylonitrile membranes were also synthesized by an electrospinning technique [65]. The amidoxime moiety was attached to silver ions after polyacrylonitrile was modified with hydroxylamine and immersed in silver nitrate aqueous solution. This membrane was used for the complete removal of *E. coli* with an efficient filtration rate of 8.0 mL/cm^2^ min. Another paper reported the preparation of boehmite nanoparticles with polyacrylonitrile [85]. The boehmite nanoparticles were synthesized through the sol–gel method. Then, these boehmite nanoparticles were immobilized on polyacrylonitrile nanofibers through the electrospinning method. The synthesized nanofibers were more effective in the removal of *E. coli* bacteria. Moreover, removal efficiency increased from 72.33% to 97.37% with an increase in boehmite nanoparticle concentration from 0 to 10% by weight. Maximum bacterial elimination was due to attractive forces among nanoparticles and bacteria, and due to the increased surface area of the nanoparticles. *E. coli* bacterial extraction from the water bodies increased to 99.99% by repeating the filtration technique four times. The adsorption value of PAN/boehmite nanofibers for cadmium ions was found to be 0.23 mg/g for a 5 mL cadmium ion solution at pH 4.0. Xiao et al. (2020b) investigated the various anionic groups of modified fibers [64] such as ethylsulfate-functionalized fibers, ethylsulfonic acid-functionalized fibers, benzoic acid-functionalized fibers, and phenol-functionalized fibers, all of which are promising adsorbents for cationic dyes. Brilliant cresyl blue, a hazardous cationic dye, was chosen to study the adsorption ability of the above modified fibers. It was found from the results that unmodified polyacrylonitrile fibers were not able to remove brilliant cresyl blue dye, while the phenolate immobilized fibers exhibited the strongest adsorption. Moreover, the adsorption values of the sorbents were decreased in the basic medium. Chen et al. (2021) reported the triazine-modified dendrimer introduced into polyacrylonitrile fibers by a grafting technique and used them for the extraction of Hg^2+^ ions from aqueous solution at adjusted pH 6 [86]. The equilibrium adsorption time was found to be 180 min. The extraction of mercury ions changed with the change in concentration from 0.1 g/L to 1.0 g/L, and adsorption did not increase beyond 1.0 g/L. Regeneration was partially achieved with 0.5 M HCl (37.22%) and 0.5 M HNO_3_ (60.36%), while 0.5 M Na_2_S resulted in 80% desorption of the adsorbent. 

Another author has discussed the synthesis of polyacrylonitrile nanofiber mat by an electrospinning technique and embedded α-Fe_2_O_3 _into nanofibers through a hydrothermal process [87]. This α-Fe_2_O_3_/polyacrylonitrile nanofiber mat could effectively extract lead ions from the mixture of nickel and copper ions at pH 4.8. A pseudo-second-order model better explained the kinetics of adsorption. Moreover, the Langmuir adsorption isotherm better explained the adsorption process than the Freundlich isotherm. Haddad and Alharbi (2019) reported the incorporation of various quantities of zinc oxide into polyacrylonitrile nanofibers and utilized them for the extraction of Pb^2+^ and Cd^2+^ ions [88]. A field emission scanning electron microscope (FESEM) was used to study the surface morphology of polyacrylonitrile nanofibers as well as zinc oxide immobilized polyacrylonitrile nanofibers. The addition of zinc oxide on the surface of the nanofibers increased the mean diameter from 135 nm to 240 nm. Z. Xu et al. (2018) fabricated TiO_2-_Ag_3_VO_4 _polyacrylonitrile nanofibers through an electrospinning process and used them to remove methylene blue [89]. The extraction of dyes increased within the pH range of 3-8 and further, there was no change in removal efficiency with an increase in pH. The adsorption process was also affected by temperature, contact time, and adsorption dose. Morillo Martn et al. (2018) reported effective ion-selective polyacrylonitrile nanofibers that were chemically modified in two steps: first, hydrolysis, and then reaction with ethylenediamine (EDA), ethyleneglycol (EG), or diethylenetriamine (DTA). These chemically modified nanofibers were used to extract Cu^2+^, Pb^2+^, and Zn^2+ ^ions from aqueous solutions [90]. The anions such as Cl^−^, NO_3_^2−^, SO_4_^2−^, and PO_4_^3− ^present in water were found to affect the adsorption process by competing with the metal ions and hence lowering the adsorption ability towards the adsorbent. In another study, the authors used an electrospinning technique to create a different amine-modified polyacrylonitrile nanofiber to extract anionic Congo red dye from aqueous solution [91]. Since Congo red dye is an anionic dye, it could be easily attracted by cationic species (-NH_3_^+^) present on the surface of the adsorbent in acidic conditions. Sun and Zheng (2020) reported polyacrylonitrile anion exchange fibers immobilized with polyethylenimine [92]. Alkylation of amine moieties present in polyethylenimine-functionalized polyacrylonitrile was carried out to synthesize three anion exchange fibers (PAN-PEI-3C, PAN-PEI-5C, and PAN-PEI-8C). These three adsorbents were studied for the removal of nitrate ions. It was found from the results that the uptake values of all adsorbents were the same, but PAN-PEI-5C showed more attraction towards the nitrate ions. Zhao et al. (2017) reported polyacrylonitrile fibers synthesized through an electrospinning process and covered with carbon matter via an aqueous carbonization method utilizing diethylenetriamine and glucose as the forerunner [93]. Here, toxic metal ions, chromium (Cr^6+^), and herbicide 2, 4-dichlorophenoxyacetic acid were removed through the obtained resin. Chromium was removed in two steps: (1) Cr^6+^ diffused in the form of HCrO^4−^ and Cr_2_O_7_^2−^, which were attracted by the -NH_3_^+^ moiety of amine-containing hydrothermal carbon-coated polyacrylonitrile fiber (PAN@NC) and (2) Cr^6+^ was reduced to Cr^3+^ ions by gaining electrons from the -NH_3_, -OH, and -COOH moieties. Elzain et al. (2019) synthesized carbon nanotubes and graphite impregnated poly(styrene–acrylonitrile) nanofibers to adsorb methylene blue dye [94]. A precipitation technique was used to make these nanoparticles utilizing potassium persulfate as an initiator, and then poly(styrene–acrylonitrile) poly(St-co-AN) was electrospun into nanofibers through an electrospinning method. The Langmuir adsorption isotherm was followed by poly(St-co-AN) nanofibers during the adsorption of methylene blue. Moreover, the rate of adsorption was well explained by a pseudo 2nd order model.

In another experiment, a chitosan immobilized polyacrylonitrile nanofibrous mat was prepared, as chitosan could easily bind to the nanofibers due to hydrogen bonding [95]. The mat was used to check the removal efficiency of acid blue-113 anionic dye. This adsorbent could be regenerated five times with almost similar efficiency to remove the adsorbate. The adsorption process was basically monolayer followed by the Langmuir adsorption isotherm. Mokhtari-Shourijeh et al. (2020) synthesized electrospun porous polyacrylonitrile/polyvinylidene fluoride nanofibers to adsorb direct red 23 dyes [96]. The resultant nanofiber porosity and diameter could be increased by dissolving them in a solvent and then removing the solvent to enhance the fiber porosity. The adsorption phenomenon was well explained by intraparticle diffusion kinetics and the Langmuir isotherm.

Polyacrylonitrile-coated graphene/carbon nanotube hybrid beads were prepared to absorb methylene blue dye [97]. During the synthesis of this adsorbent, the first step was the formation of a hydrogel through emulsification and self-assembly of graphene sheets and carbon nanotubes, and then immobilizing the polyacrylonitrile onto the hydrogel. In the second step, this polyacrylonitrile-coated hydrogel was activated through a potassium hydroxide carbonization technique. Through this whole procedure, extremely porous graphene/CNT hybrids were obtained with high mechanical strength and better physical properties. Yarandpour et al. (2018) synthesized core–shell nanofibers using a mixture of polyacrylic acid and dextran-polyaniline via electrospinning and in situ polymerization techniques, and used the formulated nanofibers to absorb Pb^2+^ and Cu^2+^ ions [98]. Polyacrylic acid and dextran were connected through crosslinking using a thermal treatment technique. Since dextran is a water-soluble polysaccharide and has many side chains, it can be easily chemically modified by aldehydes, methacrylate, thiol, phenol, maleimide, and vinyl sulfone moieties. Calcium carbonate was incorporated in various amounts into nanofibers of polyaniline to make the structure mesoporous. Nasimi, Baghdadi, and Dorosti (2020) prepared ethylenediamine-modified polyacrylonitrile fibers for the removal of mercury ions from aqueous systems [99]. The Freundlich, Langmuir, UT, Redlich-Peterson, and Temkin isotherm models were used to explain the equilibrium adsorption value of mercury ions by the above adsorbent. Box-Behnken designs in combination with response surface methodology were employed to explain the factors affecting the adsorption process, such as pH, equilibrium time, temperature, and initial concentration. A batch test as well as a column method were used to study the various parameters affecting the adsorption process. The Langmuir adsorption isotherm was found to explain the adsorption process well. Phan et al. (2020) prepared a composite by impregnating zinc oxide nanoparticles onto polyacrylonitrile nanofibers [100]. The addition of hinokitiol to composite nanofibers was found to be effective in increasing nanofiber efficiency. It also improved antibacterial activity against bacteria such as *Escherichia coli* and *Staphylococcus aureus*. Hinokitiol is related to terpenoid compounds obtained from the wood of trees in the family Cupressaceae. Abd-Elhamid et al. (2019) fabricated a composite of graphene oxide/polyacrylonitrile/β-cyclodextrin nanofibers by an electrospinning method to study their efficacy for the removal of crystal violet [101]. A light microscope was used to obtain the light image of the composite nanofiber and X-ray diffraction was used to study the crystallography of the nanofiber adsorbent. Another study reported ZnO-TiO_2_-modified polyacrylonitrile nanofiber mats prepared by electrospinning and their comparison with ZnO and TiO_2_ polyacrylonitrile nanofiber mats for chromium ion extraction from aqueous solution. These nanoparticles were synthesized through the arc discharge method with similar spinning factors and circumstances. The sorption of chromium is affected by the pH of the solution as it exists in different oxidation states in acidic conditions such as H_2_CrO_4_, HCrO_4_^−^, CrO_4_^2−^, and Cr_2_O_7_^2−^and was carried out at pH 1.5 and 6.0 [102].

### 3.3. Polyacrylonitrile in Membranes

The extraction of the pollutants using different membranes is a significant approach in the field of chemistry. Among these different membranes, ion exchange membranes are mostly used in fields such as dialysis, reverse osmosis, food industries, wastewater remediation, pharmaceutical industries, etc. Polyacrylonitrile, when used as a membrane for various processes, showed outstanding features, including heat resistance, and tolerance towards solvents, microorganisms, and radiation [103]. Khan and Baig (2012) reported an anion-exchange membrane of polyacrylonitrile/silica gel prepared through the solution casting method by taking various concentrations of polyacrylonitrile and silica gel [104]. The maximum anion exchange value was obtained with an equal ratio of polyacrylonitrile and silica gel. The above membrane was used to manufacture an arsenic selective electrode to determine the different concentrations of arsenic in solutions. The useful pH of the electrode was 5.0–10.0. In addition, the electrode demonstrated good selectivity for As^5+^ over interfering anions. In another study, highly porous polyacrylonitrile nanofibrous membranes were successfully fabricated using a hot water bath as an extractor from a polyacrylonitrile and poly(vinyl pyrrolidone) (PVP) blended solution, and then aminated with diethylene triamine (DETA). The obtained aminated nanofibrous mats (APAN) showed unique micro/nano structures and possessed an extra-high extraction capability for the removal of lead ions (Pb^2+^) from aqueous solution (maximum uptake capacity of Pb^2+^ was up to 1520.0 mg/g) and could maintain over 90% of its extraction capacity at the sixth cycle of extraction–dissociation [105].

Adsorptive ultrafiltration membranes were formulated from synthesized polyvinyltetrazole-co-polyacrylonitrile (PVT-co-PAN) by nonsolvent-induced phase separation [106]. PVT-co-PAN with varied degree of functionalization was explored to prepare adsorptive membranes. It was found that PVT segments could alter the pore size, charge, and hydrophilic behavior of the membranes; the membranes became more negatively charged and hydrophilic after the addition of PVT. The PVT segments also served as the primary binding sites for Cu^2+^ ion adsorption from aqueous solution. At pH = 5, the maximum adsorption of Cu^2+^ ions in a continuous ultrafiltration solution of 10 ppm were achieved. The Freundlich isotherm model accurately described the aqueous solution Cu^2+^ ion adsorption on membranes. The adsorption capacity obtained from the Freundlich isotherm model was 44.3 mg/g, which is higher than others. Alharbi et al. (2020) produced bilayer nanofiber membranes with enhanced adsorption and mechanical properties by functionalizing a layer of polyacrylonitrile with metal oxides (ZnO or TiO_2_) and chitosan via consecutive electrospinning [107]. The incorporation of a chitosan layer into nanofibers increased lead adsorption capacity by 102% and cadmium adsorption capacity by 405%. Chitosan also enhanced the tensile strength of the membrane by approximately 68%, owing to the strong interaction between the fibers at the interface of the two layers. A. Almasian et al. (2018) prepared a polyacrylonitrile/polyaniline (PANI)-nylon core–shell nanofiber membrane as a filtration–adsorption membrane and modified its surface with diethylenetriamine (DETA) for separating Pb^2+^ and Cd^2+^ ions from aqueous solutions [20]. The modification resulted in an increase in the hydrophilicity and permeability of the membrane. The anti-fouling of the membrane was tested by bovine serum albumin filtration. The results revealed a rejection efficiency of 89.11% for BSA and a flux recovery ratio of 91.85%, which can be owed to the hydrophilic nature of nanofibers. The prepared membrane also showed an adsorption capacity of 960 mg/g and 911.72 mg/g for lead and cadmium ions, respectively, due to incorporated nitrogen atoms on the nanofiber surface. The use of nylon as the core significantly improved the mechanical properties of the membrane. The results showed the rejection of 96.77% and 95.11% for Pb^2+^ and Cd^2+^, respectively, for 12 h. The modified membrane also showed a good regeneration property, and the rejection of Pb and Cd ions was 87.57% and 86% even after 10 filtration cycles. Another paper described the preparation of a membrane of polyacrylonitrile and its corresponding membrane coated with polyaniline (PANI) for the adsorption of heavy metal ions Pb^2+^ and Cr_2_O_7_^2−^. Membranes coated with PANI showed better adsorption performance and their direct current conductivities were correlated to the concentrations of heavy metal ions. The maximum adsorption capacities of lead and chromate ions on the PANI-coated membranes were found to be 290.12 mg/g and 1202.53 mg/g, respectively [108]. Xu et al. (2020) selected graphene oxide as a structural optimizer to tune the porous structure of the polyacrylonitrile membrane [109]. The pore size and porosity of the membrane were increased from 156.4 nm and 37.0% to 590.8 nm and 81.4% with an increase in graphene oxide amount from 0 to 0.15%. The membrane was then decorated with well-dispersed iron/manganese oxide to achieve a functional membrane that could efficiently remove methylene blue from water in the presence of peroxide. The functional membrane was found to have a higher oxide decoration ratio, lower water contact angle, larger pores, higher porosity, and better mechanical properties compared with the membrane without graphene oxide. The solution flux of methylene blue was approximately two times higher. Moreover, the functional membrane could remove the dye from water even after 30 cycles without any obvious efficiency attenuation, while the removal efficiency of the membrane without graphene oxide was clearly attenuated after 25 cycles. In another study, polyacrylonitrile nanofiber membrane was prepared by electrospinning and was functionalized with hydroxylamine hydrochloride and sodium hydroxide to obtain the amidoxime modified nanofiber membrane [110]. The amidoximated polyacrylonitrile nanofiber membrane was then kept in CuSO_4_ and FeCl_3 _metallic solutions to produce Cu-Fe bimetal-improvised polyacrylonitrile nanofiber membranes (BM-PANNM). This adsorbent was used to remove reactive blue 19. It was found that a copper-loaded membrane showed slower degradation than an iron-containing nanofiber membrane. R. Wang et al. (2013) synthesized a new membrane by fusing cellulose nanofiber (CNF) into a bilayer of nanosize electrospun polyacrylonitrile/microsize polyethylene terephthalate [111]. The resultant membrane was efficient at removing bacteria, viruses, and hazardous metal ions.

### 3.4. Polyacrylonitrile in Hydrogels

**Figure 7:**
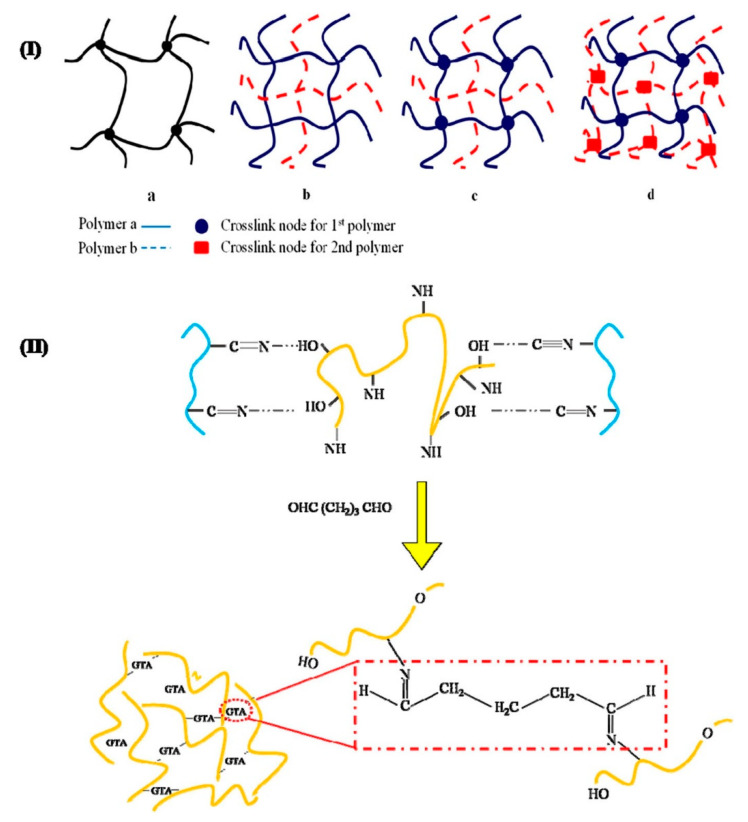
(**I**) Polymer hydrogels: (**a**) polymer network, (**b**) polymer blend, (**c**) sIPN, (**d**) full IPN, and (**II**) schematic representation of preparation of semi-IPN. Adapted with permission from reference [22].

Hydrogels consist of a three-dimensional structure of polymer capable of absorbing large quantities of water and thus can also be used in many biomedical fields. However, hydrogels do not dissolve in water due to chemical and physical crosslinking between the chains of the polymer. Moreover, the functional groups present in hydrogels, such as COOH, -SO_3_H, -NH_2_, and -OH, improve the chelating power and increase adsorption value towards the toxic metal ions and dyes [112]. Hydrogels have attracted the attention of many researchers because of their lower cost and more effective properties [113]. Available literature confirms that hydrogels are effective in the removal of water pollutants. In one study, polyacrylamide hydrogel was synthesized using different concentrations of amine via transamidation and Hofmann methods [114]. The obtained products were utilized for the extraction of Cu^2+^ ions at pH 5.5 by the batch adsorption method. The maximum extraction value of copper was 2.93 mmol/g, acquired after a recovery cycle. Adsorption rate constants and activation energy of resin for ruthenium and iridium were also reported in this investigation. M. Ajmal et al. (2015) reported cobalt–iron bimetallic magnetic nanoparticles prepared through the inverse suspension polymerization method [115]. The amidoxime group was introduced by reacting the cyano group with hydroxylamine hydrochloride, which helped to increase the extraction capacity of the microgel. The magnetic behavior was introduced in the functionalized microgel matrix by in situ synthesis using cobalt–iron nanoparticles. This modified magnetic microgel was an effective adsorbent for the extraction of metal ions such as cadmium, chromium, and organic dyes such as methylene blue, rhodamine 6G, and herbicide paraquat. The adsorption values of amidoxime-poly(methacrylic-co-acrylonitrile) microgels for methylene blue, rhodamine 6G, paraquat, cadmium, and chromium ions were 88.1 ppm, 89.9 ppm, 190.0 ppm, 334.5 ppm, and 166.5 ppm (previous values were 40.2 ppm, 37.4 ppm, 75.3 ppm, 57.4 ppm, and 56.3 ppm). In another study, the ultra-fine CNF membrane was synthesized through a TEMPO (2,2,6,6-Tetramethylpiperidin-1-yl) oxyl) oxidation approach [116]. The dynamic adsorption experiment was performed in dead-end filtration to study the adsorption of lead and chromium metal ions. The pH of the solutions was adjusted by using sodium hydroxide solution and hydrochloric acid. The elimination of bacteria and viruses through filtration membranes was determined via a retention test. For this test, 10 mL of *E. coli* suspension (106 CFU/mL) or MS2 bacteriophage suspension (106 PFU/mL) was used in a dead-end filtration mode. Different copolymer hydrogels of acrylonitrile-*N*-vinyl-2-pyrrolidone (NVP/AN) and acrylonitrile-methylmethacrylate (NVP/MMA) were synthesized by subjecting them to γ-irradiation in different dosages. These hydrogels were then modified with a hydroxylamine–hydrochloride solution at pH 7. The amidoxime-containing hydrogels were washed to remove hydroxylamine, desiccated at 70 °C for six hours, and used to remove dyes from industry wastewater. It was found that the adsorption of dyes increased with the increase in concentration of dyes in a synthesized solution up to 200 ppm, after which there was a decrease in adsorption values. Results showed that modified NVP/AN hydrogel showed more attraction for dyes compared with NVP/MMA. Moreover, dye adsorption increased with an increase in temperature because of the heating effect. In another study, a semi-interpenetrating polymer network (semi-IPNs) was prepared by combining chitosan and polyacrylonitrile hydrogel with glutaraldehyde by a casting solution process [22]. As hydrogels are soft and can be easily distorted, semi-IPN was used to increase the stability and mechanical strength of the gel. In semi-IPNs, one polymer is crosslinked and the other is present in linear form, as shown in Figure 7. These were prepared by polymerization of acrylonitrile with chitosan and were used for the adsorption of Rhodamine B dye.

A 3D hydrogel was synthesized by immobilizing sodium alginate into graphene oxide (GO) by using ultrasonic radiation. This solution was then modified with acrylonitrile by stirring and washed with dimethylformamide to obtain the precipitate. Afterwards, calcium chloride solution was added into the resultant precipitate of the graphene oxide/alginate-polyacrylonitrile (GO/Ca-Alg_2_-PAN) adsorbent. Generally, GO is a good adsorbent due to its high stability and large surface area, but sometimes it becomes difficult to regenerate GO due to its hydrophilic nature. Therefore, sodium alginate was encapsulated into GO to convert the single 2D structure into three dimensional networks. This adsorbent was used for the extraction of copper ions from aqueous media. Dudu et al. (2015) synthesized poly(acrylonitrile-co-acrylamidopropyl-trimethyl ammonium chloride) hydrogels to remove toxic ions such as As^5+^, Cr^3+^, and Cr^6+ ^from the aqueous system [117]. The adsorbent was further treated with hydroxylamine hydrochloride (NH_2_OH.HCI) to introduce a hydrophilic amidoxime group in the hydrogels. Freundlich and Langmuir adsorption isotherms were used to study the adsorption data. As^5+^ ion adsorption followed the Freundlich model, whereas Cr^3+^ and Cr^6+^ ion data fit well into the Langmuir adsorption isotherm. Moreover, the As^5+ ^ion adsorption process followed the pseudo first-order kinetic model, while Cr^3+^ and Cr^6+ ^ion adsorption followed the pseudo second-order models. Ekebafe, Ogbeifun, and Okieimen (2012) reported the copolymer of cassava starch (mainly found in Nigeria) and acrylonitrile by grafting technique and utilized it to remove lead, nickel, and copper ions from the aqueous system [118]. Hydrolysis of the resultant hydrogel with sodium hydroxide helped to enhance the removal efficiency of toxic metal ions. Regeneration of metal and hydrogel was carried out with 2% hydrochloric acid. Lu et al. (2020) prepared a hydrogel by using amidoxime-modified polyacrylonitrile, chitosan, and graphene oxide for the extraction of U^6+ ^ions [119]. The hydrogel was obtained in the form of small beads of a diameter of 3 mm. The Langmuir adsorption isotherm best explained that adsorption took place through the formation of a monolayer of adsorbate on the adsorbent through chemical bonding, and the rate of adsorption was described via a pseudo-second-order model. The desorption process was carried out by using 0.1 M sodium bicarbonate to obtain the adsorbent and metal ions. Table 1 gives an overview of the performance of various polyacrylonitrile-based adsorbents.

## 4. Factors that Affect the Adsorption Process 

### 4.1. Effect of pH

**Figure 8:**
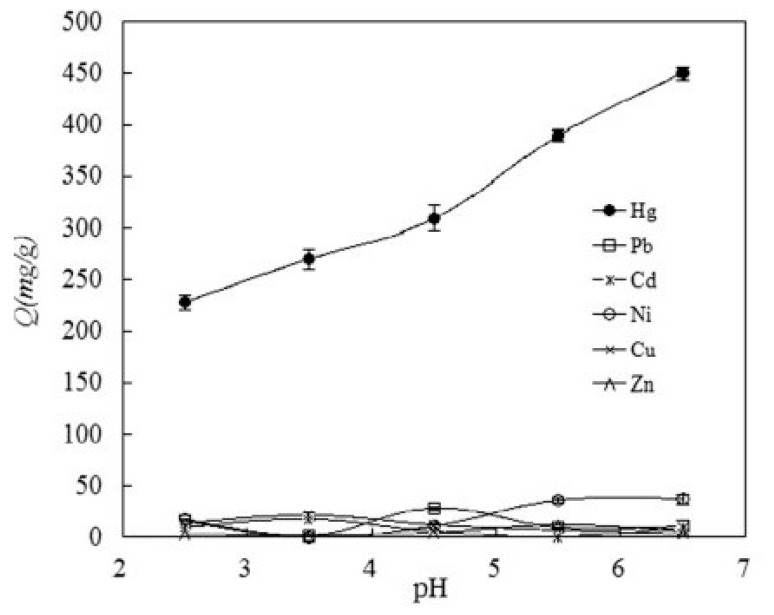
Effect of pH on the sorption value of polyacrylonitrile-2-amino-1,3,4-thiadiazole adsorbent for Hg^2+^ ions (adsorbent dose used = 0.015g, Co = 10.0 mg/30.0 mL, pH 2.5–6.5, T = 298 K, 100 rpm). Reproduced with permission from reference [45].

The extraction of the ions can be altered through the pH of the solution. The desired pH can be obtained through a suitable buffer. Buffer affects the process of ion formation during the interaction of metal ions and adsorbent. A discussion in an article explained the impact of pH on the adsorption of mercury ions by polyacrylonitrile-2-amino-1,3,4-thiadiazole (PAN-ATD) in the pH range 2.5–6.5 at 298 K. It was found that adsorption value of chelating resin rises with pH from 2.5 to 6.5 and attained the highest value 456.1 mg/g at pH 6.5. The adsorption was decreased at low pH value of solution due to the competitive adsorption effect of H^+^ ions with mercury ions. As the value of pH reached 4.0, mercuric hydroxide (Hg(OH)_2_) was the main compound to be formed. The amine group was deprotonated as the pH was increased as shown in Figure 8 [45].

### 4.2. Effect of Contact Time 

Another important parameter is the effect of contact time on the adsorption of metal ions by the adsorbent at various initial metal ion concentrations, which helps to determine the equilibrium time of adsorption. The tendency to attain the equilibrium at different times depends upon the concentration of metal ions, the adsorbent, the initial concentration, and the temperature of the solution [43,55,122].

**Figure 9:**
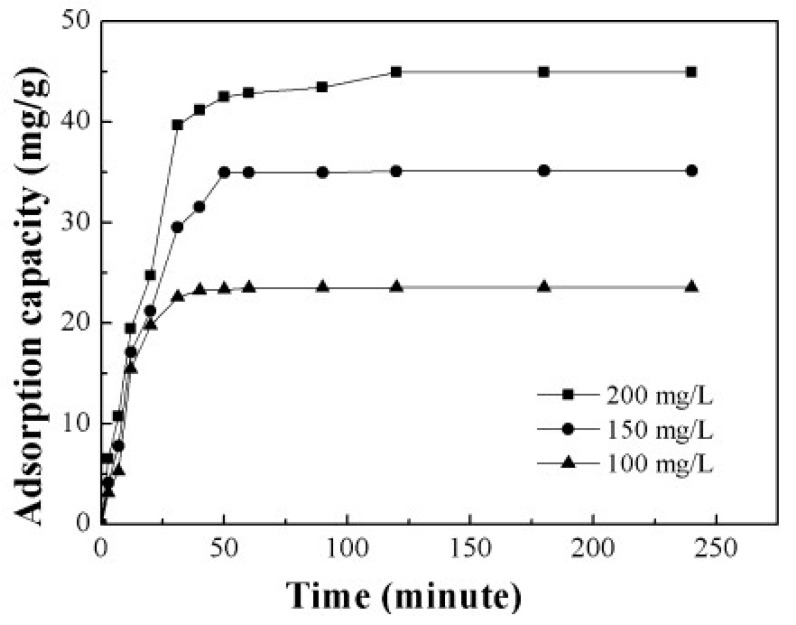
Effect of contact time and initial concentration on the adsorption of Cr^6+^ of the PAN/PPy nanofiber mat. Temperature = 25^◦^C, pH = 2.0. Adapted with permission from reference [77].

Wang et al. reported the synthesis of polyacrylonitrile/polypyrrole (PAN/PPy) core–shell structure nanofibers by means of electrospinning. The saturation of adsorption would take place within 30 to 90 min as the initial concentration increased from 100 to 200 ppm. The rate of adsorption increased with an increase in contact time; saturation values were 23.55 mg/g, 35.12 mg/g, and 44.95 mg/g at the concentrations of 100 ppm, 150 ppm, and 200 ppm of initial Cr^6+^ solution, as shown in Figure 9 [77].

### 4.3. Effect of Other Co-Existing Ions 

**Figure 10:**
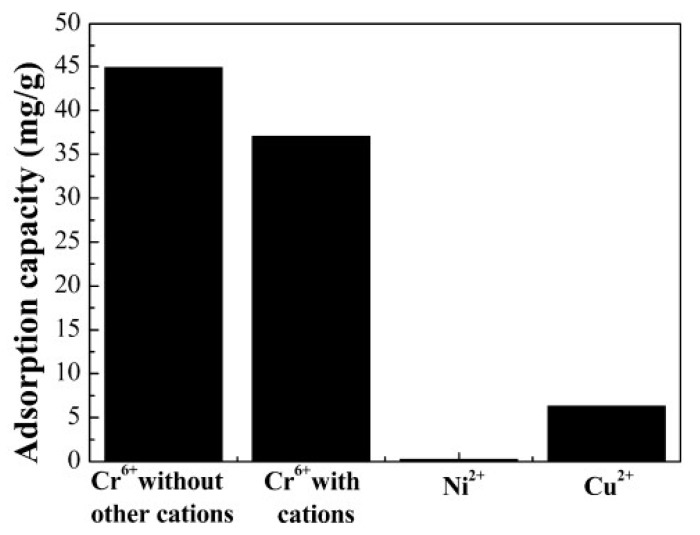
Effect of coexisting ions on adsorption capacities of PAN/PPy nanofiber mat for Cr^6+^, Ni^2+^, and Cu^2+^. Adapted with permission from reference [77].

The extraction of toxic heavy metal ions from aquatic systems is also affected by the presence of a mixture of the ions in the form of either cations or anions. These metal ions compete for their adsorption on vacant sites of the adsorbent [55,71]. In a study, the impact of coexisting ions on the adsorption process was examined in a solution containing a combination of metal ions Ni^2+^, Cu^2+^, and Cr^6+^. Adsorption by PAN/PPy nanofiber mats (100 mg) was done by dissolving the adsorbent in a 30 mL solution of a mixture of metal ions, with the initial concentration of every metal ion being 200 ppm at pH 2. It was found that uptake of Cr^6+ ^ions was decreased in the presence of Ni^2+^ and Cu^2+ ^ions (Figure 10). However, PAN/PPy nanofiber was still more selective for Cr^6+ ^ions compared with Ni^2+^ and Cu^2+ ^ions [77].

### 4.4. Effect of Adsorbent Amount

**Figure 11:**
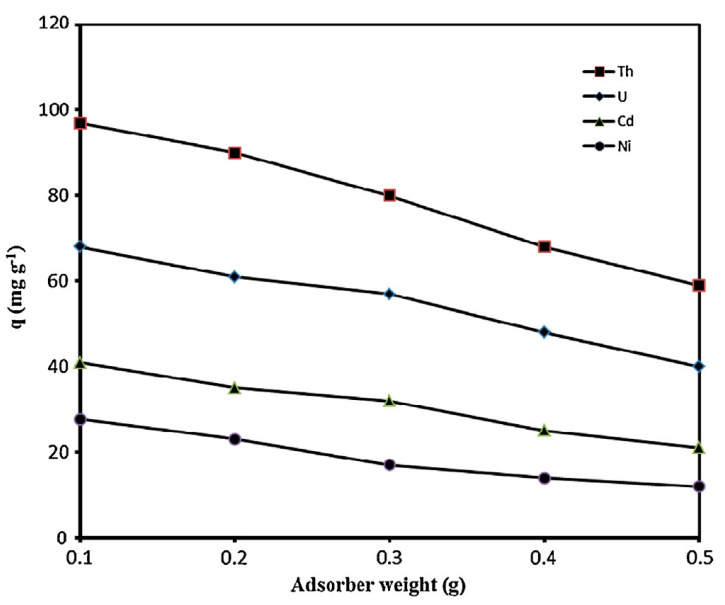
Effect of adsorbent dosage on the adsorption of metal ions. Adapted with permission from reference [129].

The amount of adsorbent is also a determining factor affecting the concentration of adsorbate adsorbed and thus is important to make the whole process of adsorption more cost-effective. The increase in adsorption efficiency with an increase in the amount of adsorbent is due to more available space for adsorption. Although, after a particular adsorbent amount, the extraction ability becomes constant. This is due to the large number of available vacant sites in comparison with the adsorbate amount. Hence, it is better to use the optimum amount of the adsorbent instead of using it in excess [45,55,128]. For example, the effect of the adsorbent amount on the adsorption of Th^4+^, U^6+^, Cd^2+^, and Ni^2+^ was examined by using an amount of 0.1–0.5 g in 100 mL of toxic metal solutions with a starting concentration of the adsorbent of 100 ppm at 298 K and pH 6.0. As evident from Figure 11, the adsorption of Th^4+^, U^6+^, Cd^2+^, and Ni^2+^ by the adsorbent was higher at 100 mg and then decreased. As the concentration of the adsorbent increased, it became difficult to approach the unused sites due to the higher concentration of adsorbent, thus reducing the adsorption efficiency of metal ions [129].

### 4.5. Reusability of Adsorbent

**Figure 12:**
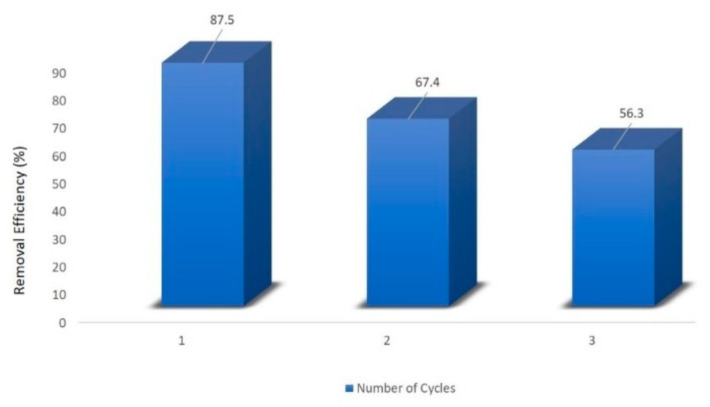
Regeneration process of the modified polyacrylonitrile. Adapted with permission from reference [128].

Revival and desorption of the adsorbent is an essential parameter for industry to reduce the cost of the extraction process. Acids (HCl, H_2_SO_4_, HNO_3_), bases (NaOH, KOH), salts (CaCl_2_, Ca(NO_3_)_2_, Na_2_SO_4_, Na_2_CO_3_), and chelating agents (EDTA) can all be used to desorb [120]. The regenerating agents are considered effective if they can recover the metal ions without destroying the adsorbent binding sites. For example, Xiong et al. (2015b) studied the desorption of mercury ions from polyacrylonitrile-2-amino-1,3,4-thiadiazole chelating resin (PAN-ATD) by using 30 mL of HCl and HNO_3_ in different amounts (0.5–4.0 M) [45]. It was found that a 4.0 M solution of HCl or HNO_3 _solution completely (100%) desorbed mercury ions. The adsorption–desorption phenomenon was carried out five times, and it hardly affected the adsorption value. Adsorption values were sustained at up to 90.8%, even after repeating this phenomenon 5 times. In one of our desorption studies, the removal efficiencies of Pb^2+^ at 0.1M HCl for cycles 1, 2, and 3 were found to be 89.3%, 63.2%, and 54.6%, respectively as shown in Figure 12 [128].

**Figure 13:**
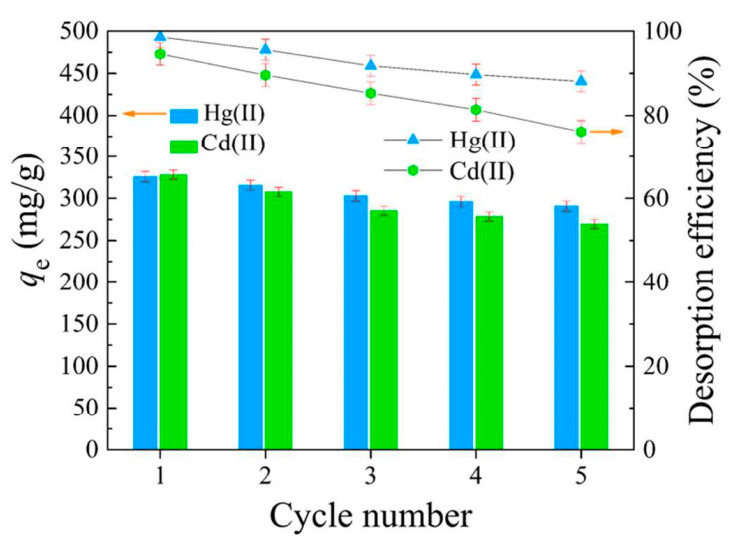
Adsorption capacities of Hg^2+^ and Cd^2+^ on PANMW-Thiol fiber regeneration. Reproduced with permission from reference [32].

Sheng Deng et al. (2017) synthesized thiol-functionalized polyacrylonitrile fibers by a microwave irradiation technique for the extraction of Cd^2+^ and Hg^2+^ ions [32]. This technique is better than conventional methods due to reduced reaction time, reduced side products formation, and an increase in the reaction yield. Moreover, the desorption of Cd^2+^ and Hg^2+^ ions was conducted through 0.1M HCl, and the regeneration process of metal ions, as well as fibers, was repeated 5 times (Figure 13). The effective desorption of these metal ions from the fibers could take place due to the greater attraction of H^+^ ions towards the fibers compared with metal ions.

### 4.6. Adsorption Mechanism

**Figure 14:**
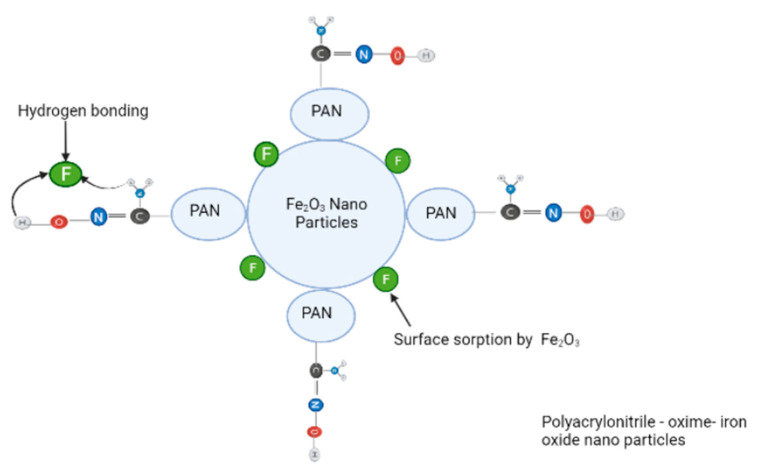
Adsorption mechanism of fluoride ions by polyacrylonitrile-oxime-iron oxide nano-particles.

Some of the methods used to remove harmful metals from the environment include surface adsorption, ion exchange, electrostatic forces, precipitation, and chelation [130]. Metal is diffused onto the adsorbent’s pores via the physisorption process known as surface adsorption. The adsorbent and the charge on the metal ions (the adsorbate) interact electrostatically to cause sorption. Moreover, the complex formation generally involves the interaction between electron donor ligands and metal ions, which is responsible for extraction. The following studies are briefly discussed:

Nouri et al. 2014 explored the properties of polyacrylonitrile/oxime/nano iron oxide as an adsorbent for the extraction of fluoride ions from aqueous solution (Figure 14) [131].

Here, the adsorption of fluoride ions occurred through hydrogen bonding, complex formation, as well by surface adsorption. 

**Figure 15:**
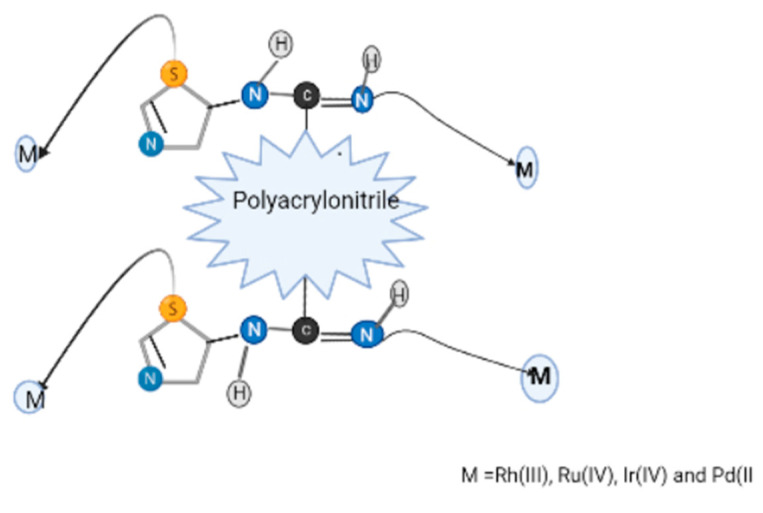
Possible sorption mechanism of different metal ions by poly(acrylonitrile-2-amino-2-thiazoline) resin. The adsorption by modified adsorbent was more selective for Pd^2+^ and Rh^3+^ in acidic conditions.

Magnificent adsorption capacity of poly(acrylonitrile-2-amino-2-thiazoline) resin was demonstrated by Y. Chen and Zhao (2003). The adsorption values of poly(acrylonitrile-2-amino-2-thiazoline) resin for Rh^3+^, Ru^4+^, Ir^4+^, and Pd^2+^ were 72.07 mg/g, 137.6 mg/g, 147.1 mg/g, and 230.7 mg/g resin, respectively (Figure 15) [132]. 

**Figure 16:**
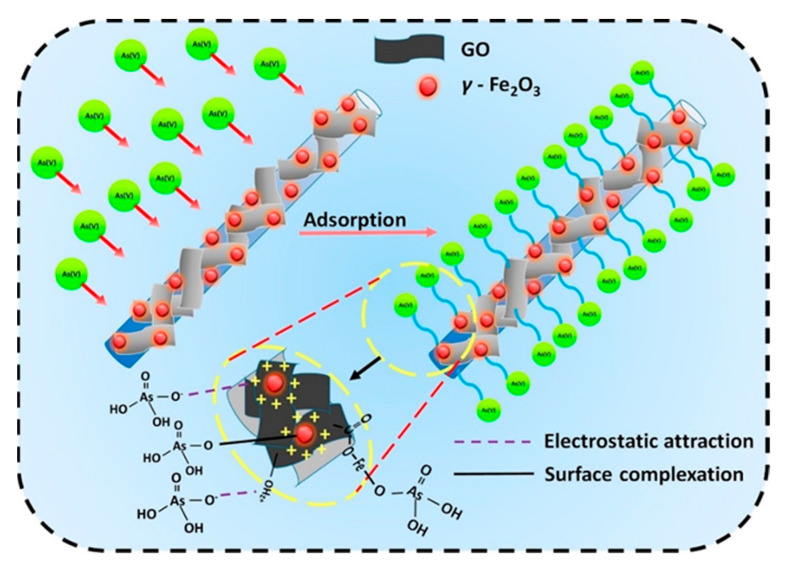
Schematic representation of possible adsorption mechanism for As^5+^ removal on the surface of PAN/GO/γ- Fe_2_O_3_ nanofiber Membrane. Reproduced with permission from reference [124].

Tripathy and Hota (2019) reported maghemite- (γ- Fe_2_O_3_) and graphene-oxide-incorporated polyacrylonitrile nanofibers synthesized through an electrospinning method and used them to remove As^5+^ ions from aqueous solution [124]. The removal efficiency was influenced by factors such as adsorbent dose (0.01–0.09 g), pH (3–8), exposure period (30−180 min), and initial As^5+^ ion concentration (1–50 ppm). As shown in Figure 16, the adsorption of arsenic by the GO/γ-Fe_2_O_3_-modified polyacrylonitrile was increased due to ionic interaction as well as complex formation by the ions.

## 5. Adsorption Isotherms Models

The relationship between the amount adsorbed and the concentration of solution is known as the adsorption isotherm. This adsorption isotherm explains the distribution of metal ions on the surface of an adsorbent as well as in solution and helps to study the efficiency of adsorbents. A variety of adsorption isotherm models are accessible to explain the equilibrium of adsorption, including Langmuir, Freundlich, BET, Toth, Temkin, Redlich–Peterson, Sips, Frumkin, Harkins–Jura, Halsey, Henderson, and Dubinin–Radushkevich isotherms [133]. A huge literature is available to show that Langmuir and Freundlich isotherms are used most of the time for extraction of toxic metal ions [44,47,134]. These are briefly described below.

### 5.1. Freundlich Adsorption Isotherm Model 

In the Freundlich isotherm model, maximum adsorption takes place on the surface of an adsorbent by forming multiple layers. This isotherm is related to heterogeneous surfaces. It is assumed that multiple layers are formed on the surface of the adsorbent, and solute particles are constantly bound to the surface of the adsorbent.

This isotherm can be described by the equations given below.

q_e_ = K_f_ C_e_^1/n^ (1)

Linear equation;

logq_e_ = logK_f_ + logC_e_ (2)

The quantity of solute adsorbed onto the adsorbent at adsorption equilibrium is represented by q_e_ (mg/g, mmol/g); C_e_ (mg/L, mmol/L) is the solute concentration in solution at adsorption equilibrium; K_f_ (mg/g) indicates increased adsorption ability of the adsorbent; and n (L/mg) is the Freundlich isotherm constant. The value of 1/n indicates the feasibility of the process. If its value is less than 1, it means adsorption is chemical; and if its value is more than 1, it indicates the process of adsorption is physical, if 1/n =0, it means the process is irreversible.

The limitation of this model is that it is applicable over a certain range of pressures and concentration only. This model fails when the concentration of the adsorbent is very high. It is a purely empirical model without any theoretical basis [135].

### 5.2. Langmuir Adsorption Isotherm Model 

A Langmuir adsorption isotherm model is utilized to explain the equilibrium between an adsorbate and an adsorbent system when the surface of the adsorbent is homogeneous. It also indicates that the adsorption is monolayer as the adsorbent has equal affinity for all adsorbate molecules [134]. Adsorption phenomena depend on the number of sites uncovered on the adsorbent, and desorption depends on the number of sites of the adsorbent covered. 

The Langmuir isotherm model is expressed as:

C_e_/q_e_ = 1/q_0_K_L_ + C_e_ q_0_ (3)

The quantity of solute adsorbed onto the adsorbent at adsorption equilibrium is represented by q_e_ (mg/g, mmol/g); C_e_ (mg/L, mmol/L) is the solute concentration in solution at adsorption equilibrium; q_o_ (mg/g, mmol/g) is the highest adsorption capability; and K_L_ (L/mg, L/mmol) is the Langmuir isotherm constant. By plotting C_e_/q_e_ versus C_e_, we obtain a linear relationship. The values of both q_0_ and K_L_ can be obtained from the slope (1/q_0_) and the intercept (1/q_0_ K_L_), respectively. 

The advantage of this model is that the surface of the adsorbent is uniform, and there is no interaction among adsorbates. It follows Henry’s law at low concentration levels. Moreover, the adsorption process is unimolecular, and when the whole surface is covered by solute, further adsorption does not occur, and this indicates a maximum of adsorption saturation of adsorption. The disadvantage is that, according to Langmuir, a saturated value of adsorption is independent of temperature, but experimentally it was found that adsorption decreases with a rise in temperature. This model does not give an idea about the roughness of the adsorbent surface or heterogeneous adsorption [135]. 

### 5.3. Dubinin–Radushkevich Isotherms (D–R Isotherm)

The D–R isotherm is used to describe the mechanism of adsorption onto a heterogeneous surface by a Gaussian energy distribution [136].

The Dubinin–Radushkevich equation looks like this:

ln q_e =_ ln q_th_ − K·*ε*^2^ (4)

Here, q_e_ is the amount adsorbed at equilibrium (mg/g), q_th_ is adsorption capacity (mg/g), K is the activity coefficient related to the mean free energy of adsorption or D–R isotherm constant (mol^2^ k/J ^2^), and *ε* is the Polanyi potential or adsorption potential (kJ/mol) that is determined by

*ε* = RT ln (1 + 1/C_e_) (5)

*R, T, and Ce are universal gas constants (8.314 J/mol/K), Kelvin temperature (K), and adsorbate equilibrium concentration (mg/L) in that order. *The approach is usually applied to distinguish the physical and chemical adsorption of metal ions with their mean free energy.

Additionally, the adsorption energy E (kJ/mol) is the free energy change needed to move 1 mol of ions from the adsorbate to the adsorbent surface. This equation expresses this term.

E = 1/2 K(6)

Plotting ln q_e_ against *ε*^2^, the constants K and q_th_ can be calculated from the slope and intercept, respectively. This means that the free energy can determine the type of adsorption; if 8 < E < 16 kJ/ mol, the process is chemisorption; if it is less than 8 kJ/mol, the process is physisorption.

In one of the research articles, the authors discussed the synthesis of polyacrylonitrile nanofiber by treating it with sodium bicarbonate and examining the adsorption efficiency of the modified adsorbent for the removal of lead and nickel ions from aqueous solutions. Different isotherm models were used in this research to study whether the sorption takes place by a physical or chemical method. The authors illustrated that the Langmuir isotherm was well fitted for the lead ions, suggesting monolayer adsorption, and the Freundlich isotherm adsorption values were well-fitted for the nickel ions, suggesting multilayer adsorption. The D–R isotherm determined the mean free energy of adsorption and suggested that both heavy metal ions are physically bound to the modified polyacrylonitrile surface. The estimated mean free energy of adsorption by the D–R isotherm was, E < 8 kJ/mol, clearly showing that both heavy metal ions were physically attached to the surface of the modified polyacrylonitrile. In the literature, several investigations based on the adsorption of metal ions on modified polyacrylonitrile surfaces have been published. In Table 2, a few research datasets have been summarized.

### 5.4. Adsorption Kinetic Models 

Adsorption kinetics explains the rate of uptake of a solute from an aqueous solution onto the given adsorbent. It gives details about the rate of adsorption and factors affecting the overall rate of reaction and the mechanism used in the adsorption process. Different kinetic models such as Lagergren’s pseudo-first-order, pseudo-second-order, Elovich kinetic equation, and parabolic diffusion model are used to study adsorption kinetic phenomena. Among all the models, the rate of adsorption is generally studied using the Lagergren pseudo-first-order model and pseudo-second-order model [133].

### 5.5. Lagergren’s Pseudo-First-Order Kinetics 

The pseudo-first-order kinetic model has been mostly used to speculate on the rate of adsorption of metals, which is expressed as

dq/dt = k_1_ (q_e_ − q) (7)

q (mg/g) is the quantity of the metal adsorbed at a particular time; q_e_ (mg/g) is the quantity of metal adsorbed at equilibrium; and k_1_ (min^−1^) is the pseudo-first-order rate constant. Integrating Equation (7) by using boundary conditions q = 0 at t = 0 and q = q at t = t, then the solved Equation (8) is:

In (q_e_ /q_e_ − q) = k_1_ T (8)

Thus, the rate constant k_1_ (min^−1^) can be obtained from the graph of In (q_e_ /q_e_ − q) versus time [133].

### 5.6. Blanchard’s Pseudo-Second-Order Kinetics

The adsorption rate factors can be further studied by using Blanchard pseudo-second-order kinetics, which is explained by

dq/dt = k_2_ (qe − q)^2^ (9)

q (mg/g) is the quantity of metal adsorbed at a particular time; qe (mg/g) is the amount of metal adsorbed at equilibrium; and k_2_ (g/mg.min) is the pseudo-second-order rate constant [133].

Arranging the variables in Equation (9) gives:

dq/(qe − q)^2^ = k_2_ dt (10)

Integrating Equation (10) using boundary conditions t = 0 to t = t and q = 0 and q = q_e_ gives:

t/q = 1/k_2_ q_e_^2^ + 1/q_e_ t (11)

A graph between t/q versus t gives the value of the constant k_2 _(g/mg h) and q_e_ (mg/g) can also be calculated.

## 6. Adsorption Thermodynamic Study

Adsorption thermodynamics are discussed in detail in order to demonstrate the thermodynamic viability of any adsorbent for metal ion adsorption. Thermodynamic parameters help to describe the energy changes involved in the adsorption process. It helps to determine whether adsorption phenomena are physical or chemical, spontaneous, or non-spontaneous, and exothermic or endothermic. Thermodynamic factors such as change in enthalpy (ΔH°), change in entropy (ΔS°), and change in Gibbs free energy (∆G^0)^ can be calculated through the Van ’t Hoff equation. The value of ∆G^0 ^is negative at a particular temperature, which indicates the adsorption process is spontaneous, and the positive change in value of ∆G^0^ suggests that desorption may take place at high temperatures. The positive value of ΔH° suggests that the reaction is endothermic, and the negative value of ΔH° suggests that the process is exothermic. A positive ΔS° implies enhanced randomness at the adsorbent/adsorbate interface.

d(InK_eq_)/dT = ∆H/RT^2^ (12)

{\displaystyle K_{eq}}K_eq_ = q_e_/C (13)

K_eq_ is the thermodynamic equilibrium constant; R is the ideal gas constant; ∆H is the reaction enthalpy; and T is the temperature (K). 

The free energy change in the reaction can be calculated by the following equation:

∆G^0^ = ∆H^0^ − T∆S^0^ (14)

The Gibbs free energy change is related to the equilibrium constant by the equation:

∆G^0^ = −RT InK_eq_ (15)

By using Equations (14) and (15), Equation (16) is obtained that shows a relationship between the equilibrium constant of thermodynamic, change in enthalpy, and entropy as explained by the Van’t Hoff equation given below.

In K_eq_ = − ∆H^0^/RT + ∆S^0^/R (16)

**Figure 17:**
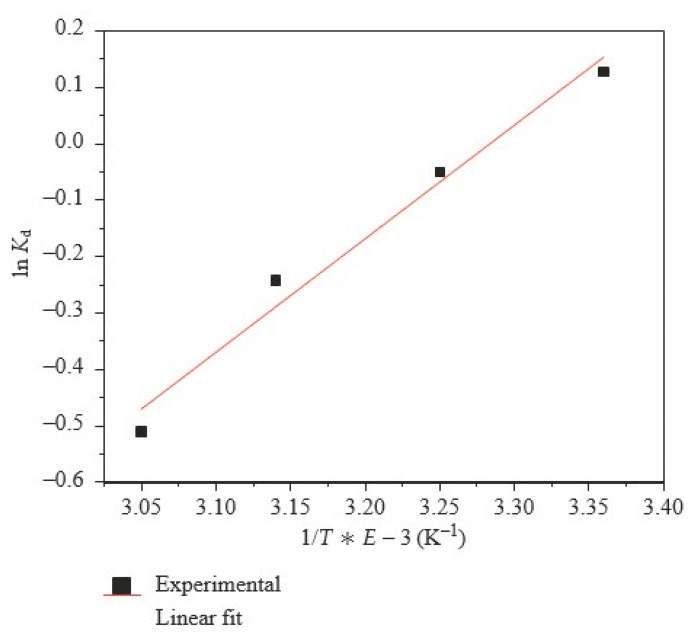
Van ’t Hoff graph for the sorption of malachite green on thiourea-modified-poly(AN co-AA) (malachite green: 100 ppm; adsorbent dose: 500 mg; contact time: 60 min; agitation speed: 100 rpm) Adapted with the permission from the reference [62].

The values of ΔH° and ΔS° are calculated from the slope and intercept of a straight-line graph between InK_eq_ versus 1/T as shown in Figure 17 [62].

Adeyi et al. investigated the effect of temperature on the adsorption of malachite green dye by thiourea-modified poly(acrylonitrile-co-acrylic acid) (TU-poly(AN-co-AA)) at various temperatures of 298 K, 308 K, 318 K, and 328 K [62]. The negative value of change in enthalpy (H^0^ = −17.06 kJ/mol) confirmed that the adsorption of the malachite green dye on TU-poly(AN-co-AA) was not supported at elevated temperatures. Further, the negative value of ∆G^0^ indicated that the process was spontaneous.

## 7. Conclusions

Water resources have consistently been polluted with industrial waste and other contaminants, such as pesticides, paints, dyes, toxic metal ions, and other substances despite the fact that water is one of the most basic needs of living things and must therefore be completely hygienic. The major water contaminants are heavy metals, including mercury, arsenic, lead, cadmium, etc. These metals are so toxic that they may cause the most serious diseases, such as cancer, respiratory diseases, immunological disorders, central nervous system issues, digestive problems, and so on. It is very important to treat the water before declaring if it is fit for human consumption. Adsorption is one of the most efficient, economical, and environment friendly methods for the treatment of environmental issues. A variety of adsorbents have physicochemical properties that make them promising candidates for wastewater treatment. Among them, polyacrylonitrile is an adsorbent that has been successfully used for wastewater treatment owing to its favorable properties. Moreover, polyacrylonitrile has a significant potential for modification, which increases the efficiency of adsorption and modified polyacrylonitrile has been used as chelating resins, membranes, hydrogels, fibers, and ion exchange resins as discussed in the present review. Polyacrylonitrile-based adsorbents also exhibit good regeneration, making the whole process more economical. Additionally, an analysis of the effects of factors on the adsorption process, including pH of the solution, adsorbent dosage, temperature, time, and initial concentration of metal ions, has also been provided. It has been determined that pH is a significant parameter that significantly influences the extraction of cations from solution. The removal of metal ions was shown to be more effective at moderate pH values compared with very low or high pH. The ratio of metal to binding sites determines the adsorbent’s capacity for adsorption, which explains how much adsorbent to use. Temperature has a considerable impact on the adsorption process as well. While an endothermic adsorption process results in greater adsorption capacity at high temperatures, an exothermic adsorption process sees a drop in adsorption rate as the temperature rises. The effect of contact time demonstrated that removal is initially quick, which slows down after reaching equilibrium. Similarly, the effect of initial ion concentration showed that the adsorption capacity of the adsorbent initially rises with increasing metal ion concentration and then falls as a result of saturation of the adsorbent’s active sites.

## Figures and Tables

**Table 1:** Performance overview of various polyacrylonitrile-based adsorbents.

S. No.	Adsorbent	Maximum Adsorption Capacity/Contact Time	Intial Concentration	Optimum pH	Reference
CHELATES					
1.	Polyacrylonitrile-2-amino-1,3,4-thiadiazole	Hg^2+^: 526.9 mg/g	-	6.5	[45]
2.	EDTA-functionalized polyacrylonitrile	Ni^2+^: 305.24 mg/g Co^2+^: 88.35 mg/g	-	5.0 1.0	[43]
3.	Ammonium molybdophosphate–polyacrylonitrile	Co^2+^: 9.424 mg/g Sr^2+^: 87.62 mg/g Cs^+^: 81.069 mg/g	10 mM	5.0 6.0 7.0	[49]
4.	Polyacrylonitrile-TETA	Ag^+^: 108.14 mg/g/30 min Pb^2+^: 99.01 mg/g/30 min	110 ppm	6.0 7.0	[120]
5.	Amidoximated AN/MA	Hg^2+^: 521.534 mg/g/600 min Ag^+^:145.62 mg/g/600 min Cu^2+^: 127.727 mg/g/600 min Fe^2+^: 41.325 mg/g/600 min Pb^2+^: 12.42 mg/g//600 min	5 mM	2.0	[55]
6.	Amidoximated polyacrylonitrile	Cd^2+^: 87 mg/g/40 min Herbicide paraquat: 91 mg/g/40 min	250 ppm 100 ppm	6.0	[57]
7.	Polyacrylonitrile-based porous carbon materials	Cr^6+^: 374.90 mg/g/120 min	448.7 ppm	2.0	[59]
8.	NaOH-hydrolyzed kapok–Polyacrylonitrile nanocomposites	Pb^2+^: 78.34 mg/g	200 ppm	-	[121]
9.	Chelating polyacrylonitrile beads	Pb^2+^: 145 mg/g/180 min Cd^2+^: 156 mg/g/1800 min	50–2000 ppm	7.0	[122]
MEMBRANES					
3.	PVAm-CNF membrane	Cr^6+:^ 23.55 mg/g/30 min Cr^6+:^ 44.95 mg/g/90 min	100 ppm 200 ppm	6.0	[77]
4.	Aminated-polyacrylonitrile nanofiber membranes	Cu^2+^: 116.522 mg/g/20 min	-	6.0	[73]
5.	Bilayer polyacrylonitrile /Chitosan nanofiber membranes	Pb^2+^: 461 mg/g/5 min Cd^2+^: 389 mg/g/60 min	150–800 ppm	5.0 7.0	[107]
6.	PAN/PANI-nylon core–shell nanofiber membranes	Pb^2+^: 960 mg/g/7 min Cd^2+^: 911.72 mg/g/7 min	50 ppm	6.5	[20]
7.	PAN/PANI membranes	Pb^2+^: 290.12 mg/g/180 min Cr^6+^: 1202.53 mg/g/180 min	5–350 ppm 5–500 ppm	7.0	[108]
8.	PAN-EDA membrane PAN-NH_2_ membrane PAN-CONH_2_ membrane	Congo red dye: 90%/60 min Congo red dye: 87%/60 min Congo red dye: 82%/90 min	10–70 ppm 10–70 ppm	-	[91]
9.	Chitosan-coated polyacrylonitrile nanofibrous mat	Acid Blue-1368 mg/g/7200 min	250 ppm	-	[95]
10.	Copper-iron bimetal-modified polyacrylonitrile nanofibrous membranes	Reactive blue 19: 99.9%/60 min	6.0/ 0.20 g	-	[110]
HYDROGELS					
1.	Magnetic Co–Fe nanoparticle-containing modified microgel	Cd^2+:^ 87 mg/g/7–20 min Paraquat: 91 mg/g/30 min	100 ppm 100 ppm	6.0 -	[115]
2.	Graphene oxide/alginate-polyacrylonitrile 3D double network hydrogels	Cu^2+:^ 381.14 mg/g/147 min	2 mM	-	[123]
3.	Amidoximated poly(acrylonitrile/*N*-vinylimidazole) hydrogel	UO_2_^2+:^ 640 mg/g gel	650–1850 ppm	4.0	[18]
4.	Amidoxime p(AN-co-APTMACl) hydrogels	As^5+^: 3333.3 mg/g/ 1480 min Cr^3+^: 26.7 mg/g/720 min Cr^6+^: 1111.13 mg/g/900 min	1–5000 ppm,	3.0 5.0 9.0	[117]
5.	Cassava starch-g-polyacrylonitrile hydrogel	Pb^2+^: 72 mg/g Cu^2+^: 76.6 mg/g Ni^2+:^ 86.5 mg/g	80 ppm 124 ppm 196 ppm	8.5	[118]
FIBERS					
1.	Amidoxime-modified polyacrylonitrile fibers	Cu^2+^: 52.7 mg/g/5760 min Pb^2+^: 263 mg/g/5760 min	- 0.2 g	[67]	
2.	Hydrolyzed/thioamidated polyacrylonitrile fibers	Cr^3+^: 37.9527 mg/g/60 min Hg^2+^: 18.05 mg/g/60 min Pb^2+^: 28.56 mg/g/60 min	10–350 ppm	[72]	
3.	Polyacrylonitrile nanofibers Amidoxime polyacrylonitrile chelating nanofibers	Cu^2+^: 215.18 mg/g/4320 min Fe^3+^: 221.37 mg/g/4320 min Cu^2+^: 215.18 mg/g/4320 min Fe^3+:^ 221.37 mg/g/4320 min	-	[74]	
4	Aminated chelating fibers	Hg^2+^: 657.9 mg/g	20–2000 ppm	[68]	
5.	Aminated polyacrylonitrile nanofibrous mats	Pb^2+^: 520.0 mg/g/300 min	-	[105]	
6.	Polyacrylonitrile /boehmite nanofibers	Cd^2+^: 0.23 mg/g/60 min *E. coli* = 97.37%	5 ppm	[85]	
7.	Dendrimer-grafted polyacrylonitrile fibers	Hg^2+^: 227.64 mg /g/180 min	1–500 ppm	[86]	
8.	Thio-functionalized polyacrylonitrile fibers	Hg^2+^: 322.8 mg /g/200 min Cd^2+^: 350.6 mg /g/200 min	30–500 ppm	[32]	
9.	Maghemite- and graphene-oxide-embedded Polyacrylonitrile	As^5+^: 36.1 mg/g/90 min	1–50 ppm	[124]	
10.	α-Fe_2_O_3_/polyacrylonitrile nanofiber mat	Pb^2+^: 70.34 mg/g/120 min Pb^2+^: 49.57 mg/g/120 min	100 ppm 50 ppm	[87]	
11.	Alkylated polyacrylonitrile-PEI anion exchange fiber	NO_3_^-:^ 31.32 mg/g/10 min	10–100 ppm	[92]	
12.	Polyacrylonitrile @NC fibers	Cr^6+:^ 290.70 mg/g/2880 min 2,4-dichlorophenoxyacetic acid: 164.47 mg/g/2160 min	20–300 ppm 20–300 ppm	[93]	
13.	Amine-modified polyacrylonitrile nanofiber mats	Cu^2+^: 150.6 mg/g/600 min Ag^+^: 155.5 mg/g/300 min Fe^2+^: 116.5 mg/g/300 min Pb^2+^: 60.6 mg/g/300 min	200 ppm 200 ppm 200 ppm 200 ppm	[125]	
14.	PAA/dextran/aniline nanofibers	Pb^2+^: 951.1 mg/g/5 min Cu^2+^: 832.7 mg/g/5 min	200 ppm	[98]	
15.	Amino-functionalized polyacrylonitrile fibers	Hg^2+^: 1116 mg /g/120 min	850 ppm	[99]	
16.	Polyacrylonitrile/cyclodextrin/graphene oxide nanofibers	Crystal violet: 16.47 mg/g/10 min	25–100 ppm	[101]	
17.	Polyacrylonitrile/polyamido amine composite nanofibers	Direct red 80: 999 mg/g/5 min Direct red 23: 942 mg/g/5 min	40 ppm	[76]	
18.	Aminated PAN/PVDF composite nanofibers	Direct red 23: 573.3 mg/g/2.5–5 min	50 ppm	[96]	
19.	Carbon-dot-modified Polyacrylonitrile fiber	Methyl orange: 422 mg/g	65 μg/mL	[126]	
20.	Fe/Ag bimetallic nanoparticles immobilized on EDTA-EDA-modified polyacrylonitrile nanofibers	Methyl orange: 96%	100 ppm	[127]	

**Table 2:** Various adsorption isotherm models of the metal ion and dye sorption on modified polyacrylonitrile adsorbent.

Scheme.	Adsorbents	Metal/Dye	Isotherm	References
1	Chitosan/polyacrylonitrile semi-IPN hydrogel	Rhodamine B dye	Langmuir adsorption isotherm	[22]
2	Thiourea-modified poly(acrylonitrile-co-acrylic acid)	Malachite green	Freundlich isotherm	[62]
3	Polyacrylonitrile/polyamidoamine composite nanofibers	DR80 and DR23	Langmuir adsorption isotherm	[76]
4	Nanoporous polyacrylonitrile/calcium carbonate	DB78	Langmuir adsorption isotherm	[96]
5	Anionic functionalized polyacrylonitrile fibers	BCB	Langmuir adsorption isotherm	[64]
6	Poly(methacrylic-co-acrylonitrile)	Cd^2+^, Cr^3+^, MB, R6G, and paraquat	Langmuir adsorption isotherm	[115]
7	Modified PAN/SiO_2_ composite nanofiber	Th^4+^, U^6+^, Cd^2+^, and Ni^2+^	Langmuir adsorption isotherm	[129]
8	Poly(acrylonitrile-co-acrylamidopropyl-trimethyl ammonium chloride)	As^5+^, Cr^6+^, and Cr^3+^	Langmuir adsorption isotherm	[117]
9	Activated nanofibrous polyacrylonitrile	Ni^2+^ Pb^2+^	Freundlich isotherm Langmuir adsorption isotherm	[136]

## Data Availability

Not applicable.
